# Aquaporin-7: A Dynamic Aquaglyceroporin With Greater Water and Glycerol Permeability Than Its Bacterial Homolog GlpF

**DOI:** 10.3389/fphys.2020.00728

**Published:** 2020-06-30

**Authors:** Fraser J. Moss, Paween Mahinthichaichan, David T. Lodowski, Thomas Kowatz, Emad Tajkhorshid, Andreas Engel, Walter F. Boron, Ardeschir Vahedi-Faridi

**Affiliations:** ^1^Department of Physiology and Biophysics, School of Medicine, Case Western Reserve University, Cleveland, OH, United States; ^2^Department of Biochemistry, Center for Biophysics and Quantitative Biology, and Beckman Institute for Advanced Science and Technology, University of Illinois at Urbana-Champaign, Urbana, IL, United States; ^3^Department of Nutrition, School of Medicine, Case Western Reserve University, Cleveland, OH, United States; ^4^Department of Pharmacology, School of Medicine, Case Western Reserve University, Cleveland, OH, United States; ^5^Department of Medicine, School of Medicine, Case Western Reserve University, Cleveland, OH, United States; ^6^Department of Biochemistry, School of Medicine, Case Western Reserve University, Cleveland, OH, United States

**Keywords:** AQP7, atomic structure, glycerol facilitator, molecular dynamics simulation, *Xenopus* oocytes, osmotic water permeability, glycerol fluxes

## Abstract

*Xenopus* oocytes expressing human aquaporin-7 (AQP7) exhibit greater osmotic water permeability and ^3^H-glycerol uptake vs. those expressing the bacterial glycerol facilitator GlpF. AQP7-expressing oocytes exposed to increasing extracellular [glycerol] under isosmolal conditions exhibit increasing swelling rates, whereas GlpF-expressing oocytes do not swell at all. To provide a structural basis for these observed physiological differences, we performed X-ray crystallographic structure determination of AQP7 and molecular-dynamics simulations on AQP7 and GlpF. The structure reveals AQP7 tetramers containing two monomers with 3 glycerols, and two monomers with 2 glycerols in the pore. In contrast to GlpF, no glycerol is bound at the AQP7 selectivity filter (SF), comprising residues F74, G222, Y223, and R229. The AQP7 SF is resolved in its closed state because F74 blocks the passage of small solutes. Molecular dynamics simulations demonstrate that F74 undergoes large and rapid conformational changes, allowing glycerol molecules to permeate without orientational restriction. The more rigid GlpF imposes orientational constraints on glycerol molecules passing through the SF. Moreover, GlpF-W48 (analogous to AQP7-F74) undergoes rare but long-lasting conformational changes that block the pore to H_2_O and glycerol.

## Introduction

In humans, 13 aquaporins (AQPs) promote whole-body water homeostasis, and osmotic balance across membranes in all major organs, blood cells, and even in organelles like mitochondria (Borgnia et al., 1999). Aquaporin-7 (AQP7)—together with AQPs 3, 9, and 10—belongs to the aquaglyceroporin subgroup of the AQP family based on sequence homology (Heymann and Engel, 1999) and permeability to H_2_O, glycerol (Ishibashi et al., 1997), urea (Ishibashi et al., 1997; Litman et al., 2009), purines, and arsenite (Borgnia et al., 1999; Liu et al., 2002; Sohara et al., 2006, 2009; Litman et al., 2009; Geyer et al., 2013). Various authors describe AQP7 in mouse and rat kidney (Ishibashi et al., 2000; Nejsum et al., 2000; Nielsen et al., 2002; Sohara et al., 2005, 2006, 2009), mouse skeletal and cardiac muscles (Skowronski et al., 2007), human, mouse and rat testes (Ishibashi et al., 1997; Nejsum et al., 2000; Saito et al., 2004), mouse and rat epididymis (Nejsum et al., 2000; Hermo et al., 2008), human, mouse and rat gastrointestinal tract (Zhu et al., 2016), and mouse developing inner ear (Miyoshi et al., 2017). In mouse and rat kidney, AQP7 is co-expressed with aquaporin-1 (AQP1) on the apical membrane of the proximal straight tubules (Ishibashi et al., 2000; Nejsum et al., 2000; Sohara et al., 2005), where AQP7 plays a minor role in H_2_O transport but a major role in glycerol reabsorption (Sohara et al., 2005). Moreover, AQP7 is abundantly expressed in human adipose tissue, where during lipolysis it mediates the efflux of newly generated glycerol (Madeira et al., 2015). Absence of AQP7 in mice leads to adipocyte glycerol accumulation, which triggers increased triacylglycerol synthesis, leading to obesity with severe insulin resistance (Hara-Chikuma et al., 2005; Hibuse et al., 2005; Rodríguez et al., 2006; Lebeck, 2014). Thus, upregulating AQP7 expression in adipocytes could be useful in treating these maladies (Verkman, 2012; Madeira et al., 2015; Mehanna et al., 2018).

Structurally, all AQPs share a tetrameric configuration with each functionally independent monomer composed of six membrane-spanning helices (H1-H6) arranged in a right-handed helical bundle. H1-H6 and two short α-helical segments (HB and HE), provide the framework for the internal monomeric pore with its selectivity filter (SF), built in AQP7 from residues F74, G222, Y223, and R229. The hallmark of AQPs is the conserved Asn-Pro-Ala (NPA) motif present at the N-termini of HB and HE (Murata et al., 2000). Uniquely in AQP7, the NPA motifs are NPS/NAA, eliminating the proline-stacking interaction characteristic of nearly all AQPs (Murata et al., 2000; Verkman, 2012; Bienert and Chaumont, 2014). Even though different, in AQP7 we refer to this site as the NPA constriction.

Functionally, the four independent monomeric pores in each AQP tetramer facilitate the osmotic passage of H_2_O and, in some AQPs, passive translocation of other small, uncharged solutes, including glycerol, urea, arsenite, NH_3_ and H_2_O_2_ (Borgnia et al., 1999; Heymann and Engel, 1999; Liu et al., 2002; Sohara et al., 2006, 2009; Litman et al., 2009; Geyer et al., 2013; Bienert and Chaumont, 2014). CO_2_ may also take this route in some AQPs (Wang et al., 2007; Musa-Aziz et al., 2009; Geyer et al., 2013).

Here we use a multidisciplinary approach to compare water and glycerol permeabilities of human AQP7 with those of its bacterial homolog, the glycerol facilitator GlpF. Physiological experiments on *Xenopus* oocytes heterologously expressing wild-type (WT) AQP7 or GlpF reveal strikingly higher water and glycerol permeabilities for AQP7. In our AQP7 structure, X-ray crystallography captures AQP7 monomeric pores in a unique closed state in which F74 and Y223 block passage of small solutes. Molecular dynamics (MD) simulations reveal a far greater flexibility of AQP7 that explains in part its far higher H_2_O and glycerol permeabilities compared to GlpF. MD simulations reveal striking F74 conformational changes that represent rapid interconversions from closed to open conformations. The comparable W48 in GlpF undergoes infrequent conformational changes that close the pore for prolonged periods, contributing to low glycerol permeability.

## Materials and Methods

### Cloning

We use gene synthesis to build the open reading frames (ORFs) for human AQP7 and *E. coli* GlpF (Genscript). The AQP7 ORF is cloned into the pIEX-Bac-3 for protein expression from Sf9 cells. Both AQP7 and GlpF ORFs are subcloned with a C-terminal mycHIS tag into the pGH19 plasmid for oocyte expression. We provide a complete description in Supplementary Material.

### X-ray Crystallography

We express protein in Sf9 cells, and purified, concentrated protein is dialyzed in 20 mM Tris–HCl pH 7.8 containing 200 mM NaCl, 2 mM β-mercaptoethanol and 0.3% decyl maltoside. Details of crystal formation and data collection/analysis are described in Supplementary Material.

### Molecular Dynamics Simulations

We use the 3.95 Å resolution crystal structure reported here as the structural model for all simulations described in Supplementary Material.

### Physiological Assays

For functional assays using *Xenopus* oocytes, we provide—in Supplementary Material: detailed descriptions of the cRNA synthesis and injection; solutions; animal procedures; swelling (including *P*_f_), uptake and surface biotinylation assays.

### Statistical Analysis

For the data presented in Figures 4, 6 and Supplementary Figure S9, we establish statistical significance by performing unpaired-samples Welch’s *t*-tests to generate unadjusted *p*-values. We set the familywise error rate (FWER) to α = 0.05 for an individual test, and apply the Holm-Bonferroni (Holm, 1979) method to control for type I errors across multiple comparisons. For all the comparisons in the test group, we order the unadjusted *p*-values from lowest to highest. For the first test, we compare the lowest unadjusted *p*-value to the first adjusted α value, α/*m* (where *m* is the number of hypotheses tested in the group). If we reject the null hypothesis, we then compare the second-lowest *p*-value to the second adjusted α value, α/*m*-1. If we reject the null hypothesis yet again, we then compare the third-lowest *p*-value to α/*m*, and so on. If at any point the unadjusted *p*-value is ≥ the adjusted α, the null hypothesis is accepted and all subsequent hypotheses in the test group are considered null.

## Results

### Physiology, I

#### Osmotic H_2_O Permeability in Cell-Swelling Assays

In our initial physiological studies, we determined the osmotic water permeability (*P*_f_) of *Xenopus* oocytes heterologously expressing AQP7 or GlpF. In both cases, these were wild-type (WT) proteins, tagged with myc-HIS. We used video microscopy to monitor rates of osmotically induced swelling, from which we computed *P*_f_ (Materials and Methods). We employed WT human AQP1 as a positive control for *P*_f_, but as a negative control for glycerol permeability.

**FIGURE 1:**
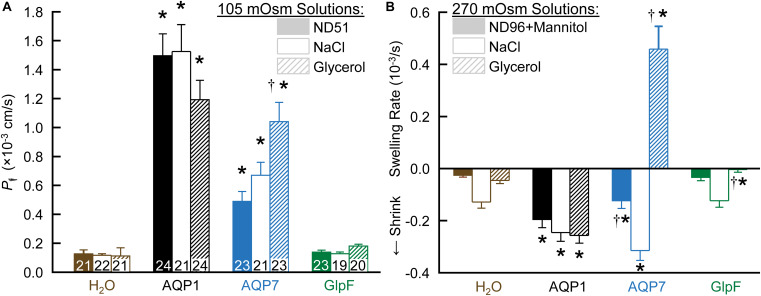
The dependence of *P*_f_ assays on solute composition in oocytes expressing AQPs. **(A)** Osmotic swelling assays. Here we lower osmolality to 105 mOsm by transferring oocytes to our ND51 solution, or HEPES-buffered solutions (pH 7.50) in which the only other solutes are NaCl or glycerol. **(B)** Osmotic shrinkage assays. After the oocytes in panel **(A)** are re-equilibrated at 195 mOsm overnight in OR3 media, we raise osmolality to 270 mOsm by transferring them to ND96 supplemented with mannitol, or to HEPES-buffered solutions (pH 7.50) in which the only other solutes are NaCl or glycerol. Bars represent mean ± S.E.M. The number of replicates are displayed at the base of each bar in panel **(A)**, and are the same in panel **(B)**. We harvested the oocytes from three frogs to perform these experiments. Statistics are performed as described in section “Materials and Methods.” The symbol “*” denotes a significant difference compared to the H_2_O control injected oocytes in the same buffer. The symbol “†” denotes a significant difference compared to both oocytes from the same cRNA injection type compared to NaCl buffer and compared to the H_2_O control injected oocytes in the same buffer.

Figure 1A summarizes data from swelling assays on H_2_O-injected controls vs. oocytes injected with cRNA encoding AQP1, AQP7, or GlpF. After pre-incubating oocytes in a normo-osmotic ND96 solution (195 mOsm), we transferred them to one of three hypo-osmotic solutions (105 mOsm): ND51 (ND96 less 45 mM NaCl), HEPES-buffered NaCl (with no other solutes), or HEPES-buffered glycerol. In each case, we video-recorded the osmotically induced swelling for 1 min and, from the swelling rate, computed *P*_f_. For both AQP1 and AQP7 oocytes, *P*_f_ values are substantially above those for H_2_O oocytes in all buffers. The ND96 and NaCl data indicate that AQP7 has substantial H_2_O permeability, though less than AQP1. Note that, for reasons that will become apparent below, the apparent *P*_f_ value for AQP7 oocytes is 55% greater with glycerol vs. NaCl as the extracellular solute.

In contrast to AQP7 oocytes, GlpF oocytes (Figure 1A, far right) exhibit *P*_f_ values that are not significantly different from those of H_2_O-injected controls for any of the three solutions.

#### Cell Shrinkage/Swelling in Hyperosmotic Solutions

After the assays in Figure 1A, we re-equilibrated the oocytes overnight in OR3 medium (195 mOsm; Musa-Aziz et al., 2010). The next day, we again pre-incubated them in normo-osmotic ND96 (195 mOsm) before transferring them to one of three hyperosmolal (270 mOsm) solutions: ND96 + mannitol, HEPES-buffered NaCl, and HEPES-buffered glycerol. In each case, we video-recorded for 1 min, anticipating to observe osmotically induced shrinkage in each case.

As summarized in Figure 1B, all three hyperosmolal solutions induce low rates of shrinkage in both H_2_O-injected controls (far left group) and GlpF oocytes (far right). Note that the shrinkage is no faster for GlpF oocytes than for H_2_O oocytes in either ND96 + Mannitol (left bar of trio) or NaCl (middle bar). Thus, under these conditions, GlpF does not display significant H_2_O permeability. On the other hand, GlpF oocytes shrink more slowly than controls in hyperosmotic glycerol (right bar of trio). We hypothesize that this slowed shrinkage reflects a modest uptake of glycerol by GlpF, followed by osmotically obligated H_2_O uptake, presumably proceeding mainly via routes other than GlpF.

For AQP1 oocytes, with their high *P*_f_, all three solutions produce a substantially faster shrinkage than for H_2_O-injected controls (Figure 1B).

For AQP7 oocytes, the results are remarkable in all three hyperosmotic solutions. Shrinkage in hyperosmotic NaCl (middle bar) is about as fast for AQP7 as for AQP1 oocytes, confirming a substantial *P*_f_. Strikingly, in hyperosmotic glycerol (right bar), AQP7 oocytes do not shrink, but swell rapidly. We hypothesize that AQP7 mediates such a large glycerol uptake as to create a paradoxical, inwardly directed osmotic gradient that produces the unexpected H_2_O uptake. In hyperosmotic ND96 + mannitol (essentially dimeric glycerol), the slowed shrinkage (left bar) presumably reflects mannitol uptake, which reduces the inwardly directed osmotic gradient.

#### Swelling in Normo-Osmotic, Glycerol-Containing Solutions

**FIGURE 2:**
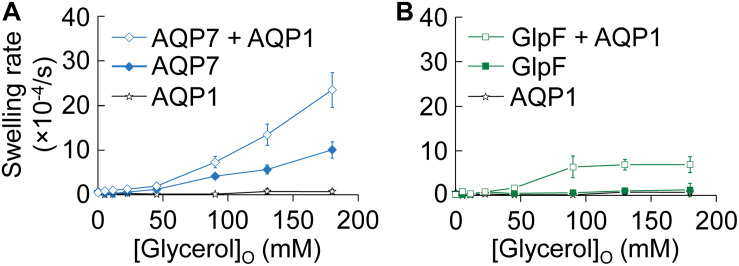
Normo-osmotic swelling of AQP7 and GlpF (± AQP1) expressing oocytes. **(A)** Oocyte swelling rates for WT AQP7 channels expressed alone or co-expressed with AQP1 as [glycerol]_o_ increased in a normo-osmotic buffer. **(B)** Oocyte swelling rates for WT GlpF expressed alone or co-expressed with AQP1 as [glycerol]_o_ in increased in a normo-osmotic buffer. All data points represent mean ± S. E. M. These data and the number of oocytes and oocyte preparations/cRNA injections used to generate each point are detailed in Supplementary Tables S1–S3.

In Figure 2, we assess glycerol permeability by monitoring oocyte swelling in normo-osmotic solutions with inwardly directed glycerol gradients of increasing magnitude. Oocytes expressing only AQP1 (Figures 2A,B, stars) do not swell because glycerol cannot enter via AQP1 (see Hub and de Groot, 2008 and Supplementary Figure S1).

Oocytes expressing only AQP7 (Figure 2A, solid diamonds), which has moderate permeabilities to both H_2_O and glycerol (Figure 1 and Ishibashi et al., 1997), exhibit modest increases in swelling rates as [glycerol]_o_ increases. Presumably, glycerol enters along its concentration gradient via AQP7, increases intracellular osmolality, and draws in H_2_O via AQP7 as well. In stark contrast, oocytes expressing only GlpF (Figure 2B, solid squares) exhibit no swelling in this assay, presumably because GlpF—although it has a small glycerol conductance—is not able to take up substantial amounts of H_2_O.

Previous work with proteoliposomes co-reconstituted with the bacterial classical aquaporin (AqpZ) and GlpF (Borgnia and Agre, 2001) has shown that the high *P*_f_ of the classical aquaporin AqpZ allows H_2_O influx to keep pace with glycerol uptake via aquaglyceroporin. Indeed, here we find that oocytes co-expressing AQP7 and AQP1 (Figure 2A, open diamonds) exhibit steep increases in swelling rate with increased [glycerol]_o_. Although oocytes co-expressing GlpF and AQP1 (Figure 2B, open squares) also exhibit increasing swelling rates with increasing [glycerol]_o_, the profile is only half as steep as that of AQP7 + AQP1 (Figure 2A), and the GlpF + AQP1 profile seems to saturate at high [glycerol]_o_ (Figure 2B).

To summarize the physiological data presented thus far, AQP7 has substantial permeabilities to both H_2_O and glycerol, whereas GlpF has an extremely low H_2_O permeability and a modest glycerol permeability. To understand the molecular basis of these striking differences, we obtained the structure of AQP7, performed MD-simulations based comparisons of AQP7 and GlpF, and then exploited these insights in further physiological experiments.

### Structural Biology

#### Crystal Packing and Overall Structure of AQP7

**FIGURE 3:**
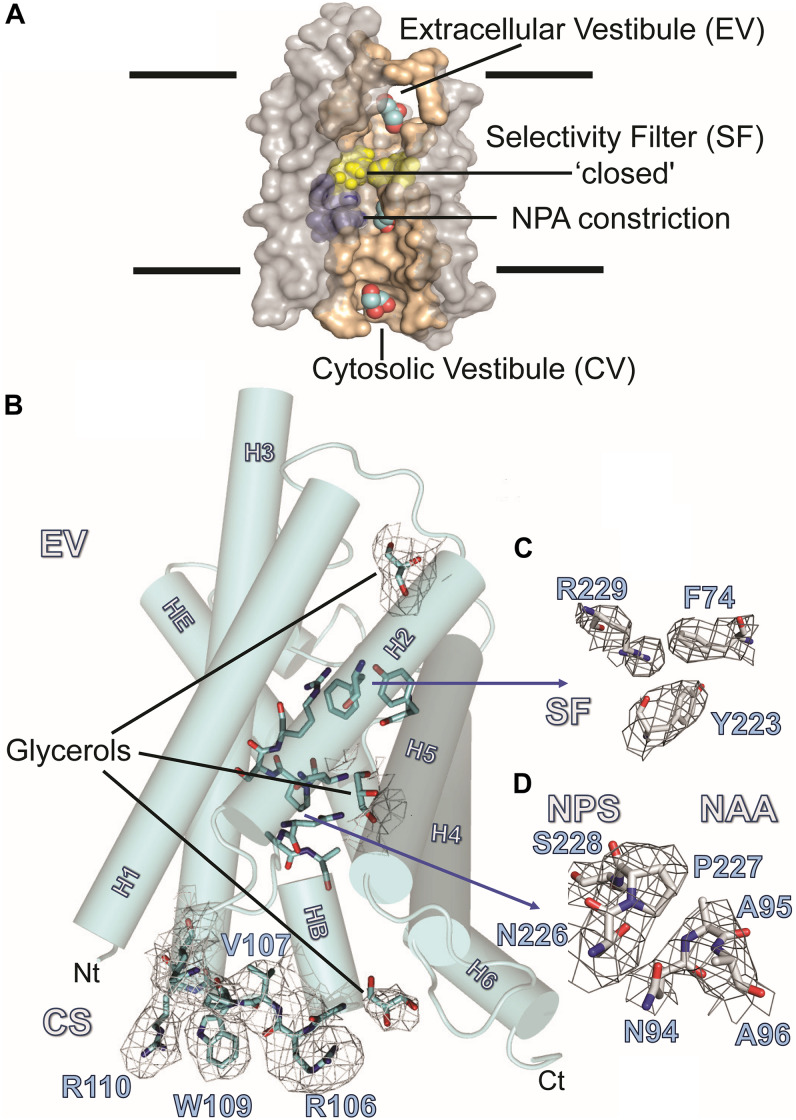
AQP7 Structure. **(A)** Van-der-Waals surface representation of the AQP7 glycerol/water channel comprising the extracellular vestibule, the closed selectivity-filter (SF, shaded yellow), the NPA constriction (shaded purple) and the cytosolic vestibule. The view into the channel has been generated by omitting half of the protein. Channel lining residues are shaded peach. **(B)** Side view of one of the four AQP7 monomers, showing glycerol channel occupancy and detailed views of the NPA (NAA/NPS arrangement) and SF region. Due to the high symmetry (F4_1_32) and the presence of non-crystallographic symmetry, side-chain electron density features are resolved at near atomic detail. Selected 2Fo-Fc maps highlight details of resolved large side chains for (i) the cytosolic loop B residues R106, V107, W109 and R110 and (ii) three glycerol molecules bound in the monomeric pore. **(C)** 2Fo-Fc map highlighting details of the SF region residues F74, Y223, and R229. Because residue G222 does not participate in the SF, it is not shown for clarity. **(D)** 2Fo-Fc map highlighting details of the NPA region residues N226, P227, S228, N94, A95, and A96. At early stages of refinement and model building, when we were employing 4-fold symmetry-averaged maps, we were able to observe a clear density, consistent with glycerol at the NPA of all 4 monomers. Thus, a glycerol is present at the NPA, albeit at low occupancy. In contrast to GlpF, no ligand electron density is observed at the SF. Two of the four monomers (including the one shown here) contain an additional glycerol molecule bound at the cytoplasmic surface (CS) region of the monomeric channel. Loop B and glycerols in panel B are contoured at 1.5 σ, SF residues in panel C are contoured at 2 σ, and NPA residues in panel **(D)** are contoured at 1.5 σ.

Figure 3A summarizes the features of the glycerol channel, comprising the extracellular vestibule (EV), the closed selectivity-filter (SF), the narrow NPA constriction, and the cytosolic vestibule. We used an AQP5 monomer as a search model to place the individual AQP7 monomers using molecular replacement techniques. Crystal packing analysis reveals 2 stacked AQP7 tetramers interfaced at the EV so that neither the N- nor C-termini participate in crystal contacts. Despite a resolution of 3.95 Å (Table 1) the high crystal symmetry (F4_1_32) and additional presence of non-crystallographic symmetry (NCS) makes it possible, in symmetry-averaged maps, to resolve electron-density features of many amino-acid side-chains as indicated in Figure 3B. Specifically, the essential SF residues (F74, Y223, and R229) are distinct (SF, Figure 3C and Supplementary Figure S2). Further examples of well-resolved residues are located at the cytosolic surface (R110, W109, V107, R106). Moreover, 2Fo-Fc simulated annealing omit maps show clear average densities consistent with glycerol in all 4 monomeric pores of AQP7. The two NCS-related AQP7 dimers comprise two monomers with 3 glycerol molecules (Glycerols, Figures 3A,B), and two monomers with 2 glycerol molecules in the monomeric pore. In all 4 monomers, glycerol molecules are resolved in the EV. Initial 4-fold symmetry averaged maps exhibited clear electron densities for glycerol ligands at the NPA constriction sites. However, the glycerol densities in the final maps are of limited quality, indicating both partial disorder and low occupancy, as documented in the PDB submission (EV, NPS/NAA, Figure 3D and Supplementary Figure S2). These glycerols at the NPA motif of our structure are consistent with those observed in the high-resolution 6QZI structure. Two monomers contain an additional glycerol molecule bound at the cytosolic surface (CS, Figures 3A,B). In stark contrast to GlpF, we observe no ligand electron density at the selectivity filter of AQP7 (SF, Figure 3C).

**TABLE 1:** 6N1G data collection and refinement statistics.

Data collection		Refinement	
Space group	F4_1_32	Resolution (Å)	32.7 – 3.95
Cell dimensions		No. reflections	18776
*a* = *b* = *c* (Å)	385.66	*R*_*work*_/*R*_free_	23.2/27.7
a = b = γ (°)	90.00	No. atoms	
Resolution (Å)	50 – 3.95	Protein	15308
*R*_pim_	0.029(0.974)*	Ligand	168
*I/*σ*I*	38.77(0.94)*	Water	n.a.
Completeness (%)	96.7(96.0)*	*B*-factors	
Redundancy	4.5(1.3)*	Protein	89.4
		Ligand	99.7
		Water	n.a.
		R.M.S deviations	
		Bond lengths (Å)	0.007
		Bond angles (°)	1.03

* Statistics for lowest resolution shell are shown in parentheses. The moderate anisotropy of the dataset significantly affects the statistics for lowest shell.

#### Glycerol in the Extracellular Vestibule

**FIGURE 4:**
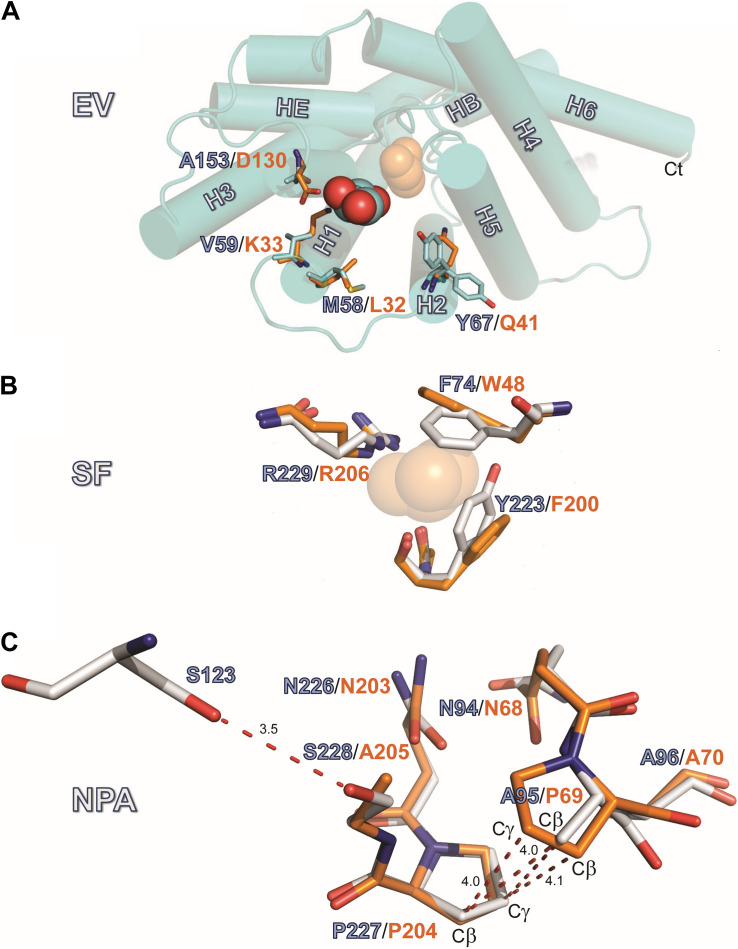
Structures of the EV, SF and NPA regions. **(A)** Location EV: View of the monomeric extracellular channel entrance of AQP7 (cyan) with a lateral shift (5.5 Å) in the position of the resolved glycerol [center: AQP7 (red-cyan), shifted right: GlpF (orange)]. We depict the narrowing of the entrance by overlaying the protruding residues of GlpF (D130, K33, L32 and Q41) and AQP7 (A153, V59, M58, and Y67). Note the inward and outward facing conformations of Y67. **(B)** Location SF: Overlay of Selectivity filter (SF) region for AQP7 and GlpF formed by two aromatic residues (Y223, F74 in AQP7), positioned at a right angle to each other, forming a hydrophobic wedge opposite from R229 and G222. G222 points away from the SF, and does not participate in the restriction. We therefore omit G222 from the figure for clarity. The glycerol molecule (transparent orange spheres) shown at this location is only present in the GlpF SF; the AQP7 SF is unoccupied. A glycerol molecule (orange spheres) at this location is present only in GlpF. **(C)** Details of the van der Waals packing arrangement of the NAA-NPS motifs involving a slight out of plane rotation of the NPS motif. The glycerol molecule at this location is not displayed in this panel for clarity and because of partial disorder and low occupancy, as documented in the PDB submission. A polar contact is observed between S228 and S123 of Helix 2. The distance unit of the annotated dashed lines is Å.

AQP7 displays a larger pore entrance at the EV than GlpF, inducing a lateral shift (∼5.5 Å) in the position of the glycerol molecule when compared to GlpF (Figure 4A). Four side chains contribute to the constriction of the entrance in both aquaglyceroporins (Figure 4A): Three of the pore-lining residues adopt an inward-facing conformation in both GlpF (D130, K33, and L32) and AQP7 (A153, V59, and M58). The fourth side chain in GlpF (Q41) is also inward-facing. In contrast, unambiguous model building shows the corresponding side chain in AQP7 (Y67) to adopt two distinct positions (inward, outward) for two of the independent monomers (Figure 4A). Interestingly, monomers with the inward-facing Y67 orientation house only two glycerol molecules, whereas monomers with the outward-facing orientation of Y67 have three glycerols.

#### Glycerol Is Excluded From the Selectivity Filter

Located halfway along each monomeric pore, the SF [also known as the aromatic residue/arginine or ar/R region (de Groot and Grubmüller, 2001)] represents the point of narrowest constriction. This constriction has a diameter of ∼2.8 Å in AQP1 (Sui et al., 2001), but ∼3.4 Å in GlpF (Fu et al., 2000), accommodating the larger size of the carbon hydroxyl group of polyols such as glycerol. As noted below, the SF of AQP7 can exist in two alternate states, one represented by our structure, with constriction diameters of only 2.1 Å or 2.2 Å in each monomer, and an alternate state revealed by MD simulations, with a constriction diameter of 3.5 Å (see Supplementary Figure S3). Similar to GlpF, where W48, G199, F200, and the highly conserved R206 define the SF, four residues also form this constriction in AQP7: F74, G222, Y223, and R229 (Figure 4B). However, the central glycerol position (G2 in GlpF; see Fu et al., 2000) is unoccupied in our AQP7 structure (SF, Figure 3). Although H_2_O would permeate through the narrow SF constriction in our AQP7 structure, the hydrophobic wedge formed by F74 and Y223 in AQP7 cannot accommodate the larger glycerol molecule. Thus, our structure represents a closed state for small solutes like polyols (Figure 4B).

#### The NPA Constriction

One of the two glycerol molecules observed in both AQP7 (Figure 4) and GlpF structures is located at the NPA constriction site, which exhibits a characteristic arrangement of two conserved asparagine residues directly forming hydrogen bonds with permeating H_2_O or glycerol molecules (Fu et al., 2000). In almost all AQPs, the prolines of the two NPA motifs stack by van der Waals interactions to form a platform for the HB and HE half helices (Fu et al., 2000; Sui et al., 2001; Gonen and Walz, 2006). Among human AQPs, AQP7 uniquely lacks the central proline in the first NPA motif. Furthermore, a serine replaces the alanine in the second NPA motif, resulting in the NAA (94–96)/NPS (226–228) arrangement. Due to these unique NPA motif modifications, the proline stacking is replaced by a different van der Waals packing arrangement, indicated in Figure 4C. Although the symmetry-averaged map of the NPA region of AQP7 is less distinct than that of the SF region (Figure 3D, NPS/NAA), the position and orientation of relevant residues were unambiguously determined. The overlay of GlpF NPA (68–70)/NPA (203–205) onto AQP7 NAA (94–96)/NPS (226–228) shows that the AQP7 NPS (226–228) motif is rotated slightly out of plane in comparison with the overlaid P69, so that the A95-Cβ is approximately equidistant between P69-Cβ and P69-Cγ (Figure 4C). As result, A95-Cβ is in van der Waals distance to both P227-Cβ and P227-Cγ of the AQP7 NPS (226–228) motif, thereby partially compensating for the loss of the proline-proline stacking. Nevertheless, the difference between these regions of GlpF and AQP7 are distinct: The buried surface area of the NPA-NPA motif in GlpF is 19.8 Å^2^, or 9.6% larger than that of the NPS-NAA motif in AQP7 (Supplementary Material). An additional structural disruption arises from the polar contact formed between S228 (of NPS 226–228 motif; see Figure 4C) and S123 of the adjacent Helix 2.

### Molecular Dynamics

#### Spontaneous Binding and Permeation of Glycerol

Based on our AQP7 structure and a previously published GlpF structure (Fu et al., 2000), we performed flooding and umbrella-sampling MD simulations that allow an examination at an atomic level of the permeabilities of AQP7 and GlpF for glycerol and H_2_O. In addition, the simulations reveal the key interactions during the passage of glycerol through the monomeric pores. For each of the two aquaglyceroporins, we performed three independent simulations (each 1,050-ns long) on membrane-embedded models of the tetrameric form, with 200 glycerol molecules initially distributed in the aqueous phase, creating an initial aqueous glycerol concentration ([glycerol]_o_) of 625 mM (Supplementary Material). During the simulations, glycerol molecules enter the monomeric pores, or penetrate into the head-group region of the lipid bilayer, from both the extracellular and cytosolic sides of the membrane. Within the first 50 ns of the simulations, the re-distribution of glycerol molecules establishes an equilibrium, with a [glycerol]_o_ of ∼500 mM.

As the simulations extend, glycerol molecules continue to enter the monomeric pores spontaneously in both AQP7 and GlpF. Regions of high-glycerol occupancy span the extracellular and cytosolic vestibules and the NPA constriction of AQP7 and GlpF. The SF is the only region with low glycerol occupancy (Supplementary Figure S1). In contrast to GlpF and AQP7, simulations of AQP1 under identical conditions show glycerol molecules diffusing only into the extracellular and cytosolic vestibules, but not entering the monomeric pore (Supplementary Figure S1).

Examination of glycerol trajectories for AQP7 and GlpF reveals that glycerol molecules enter the NPA constriction (a major glycerol binding site for both aquaglyceroporins) mostly by diffusing from the cytosolic vestibule (Supplementary Figure S4). Upon unbinding from this site, the majority of glycerol molecules diffuse back to the cytosol. Throughout the three simulations — collectively covering 3 μs of sampling—we count a total of 24 glycerol visits to the NPA site of AQP7, and 59 for GlpF. These figures correspond to average visitation rates of 2.0 ± 0.8 (AQP7) and 4.9 ± 1.5 (GlpF) per monomeric pore per μs. The smaller number of glycerol visits to the AQP7 NPA site relates to the longer glycerol residence time in AQP7 vs GlpF (see below).

The SF is the key bottleneck to translocation of the substrate through the monomeric pores. During the 3 × 1-μs flooding simulations, we observed a total of three glycerol-translocation events across the SF of AQP7 (Supplementary Figure S4A), and only one such event in GlpF (Supplementary Figure S4B). Although the number of translocation events is low, the ratio of glycerol translocations for AQP7/GlpF is in rough agreement with the physiological data in Figures 1, 2, which show that AQP7 is a much better glycerol channel than GlpF.

#### Energetics of Glycerol Partitioning Along the Pathway

**FIGURE 5:**
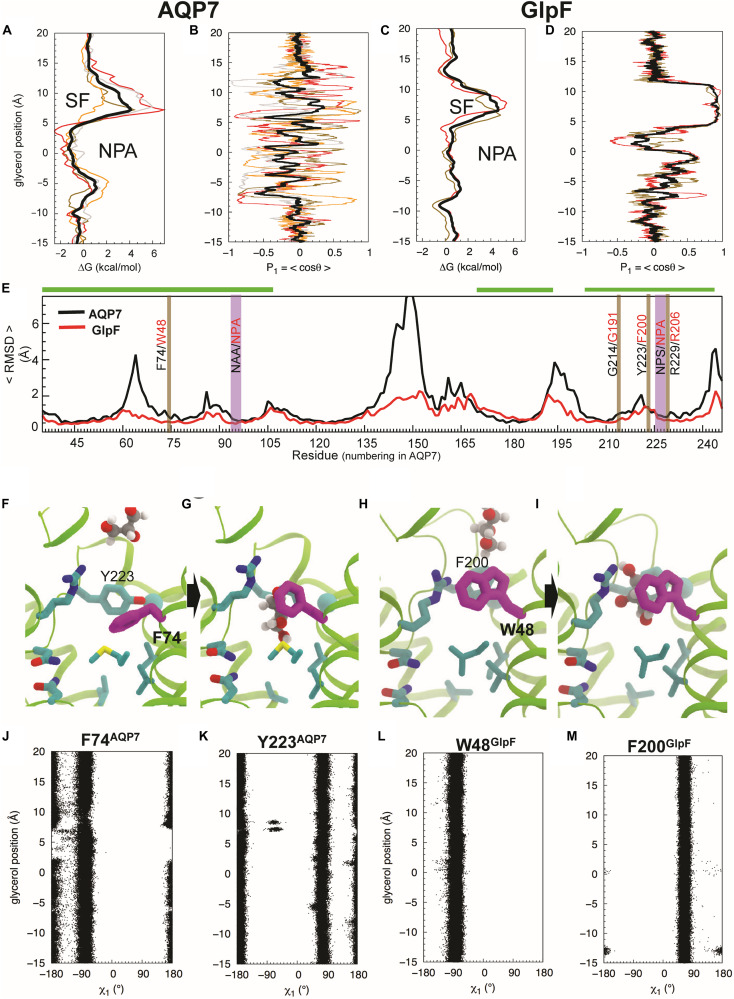
Glycerol translocation along through AQP7 and GlpF. **(A)** Free energy (ΔG) profiles of glycerol partitioning along the pores of AQP7 calculated from the umbrella sampling simulations. **(B)** Orientation (P_1_) profiles of the molecule along the AQP7 (i.e., P_1_ fluctuates between +1 and ∼ –1 and θ from ∼0° to ∼180°). **(C,D)** ΔG and P_1_ profiles of glycerol partitioning in GlpF. The profiles along the individual pores are shown as individually colored thin lines. θ is the angle between the vector connecting the first and last carbon atoms of the glycerol molecule and the membrane normal (approximately the pore axis). P_1_ is ∼ ± 1 when the orientation of the molecule is parallel to the pore axis, and <P_1_> is ∼0 when it is isotropic. **(E)** Overall conformational dynamics of AQP7 versus GlpF characterized by time-averaged RMSD profiles calculated over all monomeric channels and over all 3 flooding simulations for each protein. The NPA and SF regions are highlighted in vertical purple and brown bars, respectively. Residues of helical regions involved in glycerol permeation are highlighted in horizontal green bars. **(F–I)** MD snapshots depicting glycerol transition through the SF region. The SF is centered at *z* = ∼ 7.5 Å. F74 of AQP7 (purple) changes its conformations as the molecule transits between the extracellular vestibule and the NPA region. It is equivalent to W48 in GlpF. Also, Y223 of AQP7 is replaced by a phenylalanine (F200) in GlpF. **(J–M)** Conformations of the SF residues (F74 and Y223 of AQP7, and W48 and F200 of GlpF) as function of glycerol molecule’s position calculated from the umbrella sampling simulation, characterized by their N-Cα-Cβ-Cγ (χ_1_) dihedral angles.

We performed umbrella-sampling simulations for both AQP7 and GlpF to obtain free energy (ΔG) profiles of glycerol insertion and permeation along the monomeric pores (Figure 5A vs. Figure 5C). The SF corresponds to the section with the highest ΔG, forming a barrier of 4 kcal/mol in AQP7, and 4.5 kcal/mol in GlpF. The SF is thus the main barrier for glycerol permeation. Notably, ΔG profiles exhibit substantially greater variability among individual monomeric pores of AQP7 than GlpF (Figure 5A vs. Figure 5C). We attribute this to greater flexibility and movements in the AQP7 structure vs. GlpF (see next section and Figures 5E,J–M). In addition, a local barrier of ∼1 kcal/mol occurs in both AQP7 and in GlpF at a relative glycerol position of about –5 Å along the monomeric pore, just to the cytosolic side of the NPA site (Figure 5A vs. Figure 5C). The corresponding well at the NPA constriction of AQP7 (ΔG ≅ −1.5 kcal/mol) is deeper than that of GlpF (ΔG ≅ + 0.5 kcal/mol). Accordingly, average glycerol residence times near the NPA constriction are ∼350 ns for AQP7, but only ∼100 ns for GlpF.

#### Conformational Dynamics of Proteins and Glycerol Molecules

Backbone RMSD values for individual amino acid residues (<RMSD_res_>), averaged over all of the flooding simulations, indicate that AQP7 is conformationally far more flexible than GlpF. AQP7 regions with largest backbone <RMSD_res_> values (> 3.5 Å) include four loops (residues 61–68, 141–153, 191–202 and 241–253), whereas corresponding GlpF regions exhibit <RMSD_res_> values < 2 Å (Figure 5E). In general, AQP7 is structurally less rigid than GlpF, exhibiting higher RMSD values in most places, including AQP7 residues that interact with permeating glycerol molecules. For example, AQP7 residues near the SF and the NPA constrictions exhibit 0.1–1 Å larger <RMSD_res_> values than their equivalents in GlpF (Figure 5E).

Umbrella sampling trajectory analysis reveals that the orientation of glycerol molecules is isotropic in the aqueous solution and in most parts of the monomeric pore. We observed order parameter *P*_1_ = <cos θ> fluctuating around 0, where θ is approximately the tilt angle of glycerol with respect to the pore axis (Figure 5B; *z* > 12 Å and *z* < –8 Å). Therefore, glycerol molecules assume an isotropic orientation in both extracellular and cytosolic vestibules. In addition, no strong orientational confinement for glycerol molecules is evident during their transition through either the SF (*z* = 4 Å to *z* = 12 Å) or the NPA region (*z* = –5 Å to *z* = + 4 Å) of AQP7 (Figure 5B). In stark contrast, GlpF forces the glycerol molecule within the SF to adopt a strictly parallel orientation with respect to the pore axis, indicated by a P_1_ of ∼1 (i.e., θ≅ 0°; Figure 5D).

Orientational constraints relate to the conformations of critical residues forming the SF (Hénin et al., 2008). The equivalent residue for F74 of AQP7 (Figures 5F,G) is W48 in GlpF (Figures 5H,I). Whereas AQP7-F74 undergoes a striking conformational change in all 3 equilibrium simulations (Figures 5F,G,J), GlpF-W48 tends to maintain its X-ray–resolved conformation (Figures 4B, 5H,I,L). F74 adopts the N-Cα-Cβ-Cγ dihedral angle (χ_1_) ∼ –90° when a glycerol molecule diffuses through the SF (Figures 5G,J), which deviates largely from χ_1_ ∼ ± 180° of the X-ray structure (Figures 4B, 5F). Alignment of AQP7 and GlpF (PDB entry 1FX8) crystal structures illustrates that the X-ray–resolved conformation of F74 in AQP7 would produce a steric clash between its side chain and the SF-bound glycerol molecule in GlpF (Figure 4B). For the other aromatic SF residue in AQP7 (Y223), χ_1_ fluctuates between + 90° and ± 180° independent of the position of glycerol in the pore (Figure 5K). In contrast, both aromatic SF residues in GlpF (W48, F200) are much more stable than F74 during glycerol permeation (Figures 5L,M). The pore-lining arginine residues are even more stable in both AQP7 and GlpF (data not shown).

W48 of GlpF remains in the X-ray–resolved conformation (Figures 5H,I) except for three rare events in which its χ_1_ and χ_2_ (Cα-Cβ-Cγ-Cδ_1_) angles transit to ± 180°/–120° and ± 180°/60° (Supplementary Figure S5). These new conformations block the pore to glycerol and H_2_O. The apparently irreversible conformational transitions of W48 that we observed during the 3 × 1-μs long simulations also suggest that high-energy barrier(s) separate the X-ray–resolved and the newly observed conformations. In contrast, transitions of F74 between ± 180° and −90°χ_1_ angles occur frequently in AQP7 (Figure 5J).

#### Relations Between Glycerol Occupancy and Water Permeability

Previous MD simulations have demonstrated the H_2_O permeability of GlpF to be reduced by a factor of 7.3 in the presence of 14 mM glycerol (Chen, 2013). To explore whether AQP7 exhibits reduced H_2_O permeability in the presence of glycerol, we calculated occupancy profiles and H_2_O permeability coefficients (*P*_*d*_) distinguishing between glycerol-bound and glycerol-free (*apo*) segments of our simulations. *P*_*d*_ calculated from the 200-ns–long *apo* simulations is ∼0.16 cm/s for both AQP7 and GlpF. However, *P*_*d*_ values calculated from 250-ns–long simulations of glycerol-bound segments for AQP7 and GlpF are substantially reduced to ∼0.002 cm/s (Supplementary Figure S6A). Consistent with the calculated *P*_*d*_, we observe significant reduction of H_2_O occupancy within the pore of glycerol-bound systems, particularly at the NPA site, where the glycerol molecule is restrained by a harmonic potential (Supplementary Figures S6A–C, S7).

### Physiology, II

#### Effect of Mutations on Osmotic Water Permeability

**FIGURE 6:**
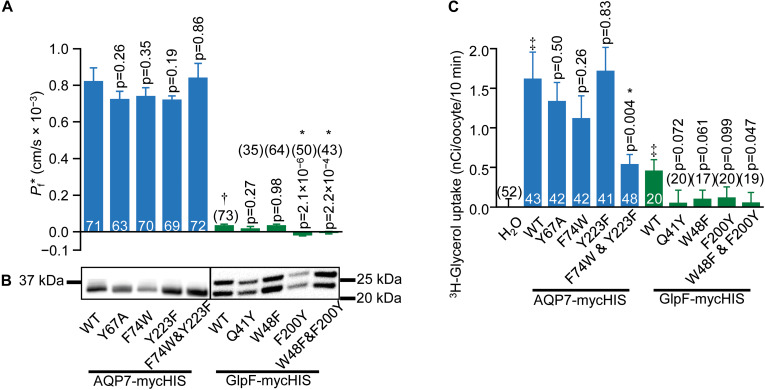
Water and glycerol permeabilities of AQP7 and GlpF. **(A)** Channel-dependent osmotic water permeability (*P*_f_*) for AQP7 and GlpF constructs. *P*_f_* for positive control AQP1 oocytes is 2.01 ± 0.10 × 10^–3^ cm/s (*n* = 81). We harvested oocytes from three frogs to perform these experiments. **(B)** AQP7 or GlpF in the surface fraction from biotinylated oocytes detected by western blot using a c-myc primary antibody. The predicted molecular weights of full-length myc-HIS tagged AQP7 and GlpF constructs are 39.8 and 32.4 kDa, respectively. AQP7 construct bands actually run in the 10% Bis-Tris gels at ∼32.5 kDa. GlpF constructs run as a doublet of 25 and 22 kDa. These smaller than expected bands reflect differing degrees of N-terminal truncation because the epitope for the primary antibody is at the C-termini of all constructs. Protein bands are aligned with the corresponding *P*_f_* bar in panel **(A)**. AQP7 and GlpF samples are analyzed on separate blots denoted by a frame around each blot image. Displayed to the side of each blot are the molecular weight markers (Other example blots are in Supplementary Figure S8). **(C)** 10-min ^3^H-glycerol uptakes for AQP7 and GlpF constructs in an ND96 solution with 90 mM total [glycerol]_0_ replacing 45 mM NaCl. For both panels **(A,C)**, bars represent mean ± S.E.M. and the number of replicates is at the base of each bar, or in parentheses above the bar. † denotes *P*_f_* for GlpF WT, which is much smaller than AQP7 WT. ‡ in panel C denotes that uptake for both AQP7 WT and GlpF WT are significantly above the H_2_O control (*p* = 3.25 × 10^–5^, and *p* = 0.014, respectively). Each *p*-value reflects comparisons of mutant to its respective WT. The symbol “*” denotes statistical significance. For statistical analyses, see section “Materials and Methods.” We harvested oocytes from two frogs to perform these experiments.

We next asked how mutations to key AQP7 and GlpF residues—those identified in the structural and MD studies—would affect *P*_f_ and ^3^H-glycerol uptake by oocytes expressing these constructs. Figure 6A summarizes the results of osmotic-swelling experiments (Supplementary Material). For all AQP7 constructs, *P*_f_ is significantly greater than H_2_O-injected control oocytes (data not shown). For AQP7-WT, the component of total *P*_f_ less the *P*_f_ of day-matched H_2_O-injected control oocytes—the channel-dependent *P*_f_ (*P*_f_^∗^)—amounts to 0.82 ± 0.07 × 10^−3^ cm/s (Figure 6A, blue bars), and is not significantly different for myc-HIS tagged vs. untagged constructs (data not shown). Both the structure (Figure 4) and MD simulations (Figure 5) suggest that the AQP7 EV residue Y67 as well as SF residues F74 and Y223 could affect glycerol and possibly H_2_O permeation. Therefore, we mutated AQP7 residue Y67 to A to broaden the extracellular vestibule, and mutated the two SF residues to match their counterparts in GlpF (W48 and F200), and *vice versa* for GlpF (Figure 6A). None of the single AQP7 mutations (i.e., Y67A, F74W, Y223F) nor even the double mutant F74W&Y223F alter *P*_f_^∗^ significantly from the value for AQP7-WT (Figure 6A, blue bars).

Oocytes expressing GlpF-WT swell at rates slightly—but significantly—greater than those of H_2_O-injected controls (Figure 6A, green bars). Thus, *P*_f_^∗^ for GlpF-WT is only 4% as large as for AQP7-WT. None of the GlpF→AQP7 mutations increase *P*_f_^∗^. In fact, both GlpF-F200Y and GlpF-W48F&F200Y reduce *P*_f_^∗^ to values indistinguishable from zero.

Surface-biotinylation experiments verify that all AQP7 and all GlpF constructs express at the oocyte surface at similar levels (Figure 6B and Supplementary Figure S8).

#### Effect of Mutations on ^3^H-Glycerol Uptake

Figure 6C summarizes, for AQP7 and GlpF constructs, ^3^H-glycerol-uptake results obtained in a normo-osmotic ND96 solution in which we substituted 90 mM glycerol for 45 mM NaCl. Glycerol uptake is significantly greater for AQP7-WT than H_2_O-injected control oocytes (blue bars). The EV mutant AQP7-Y67A does not achieve the expected higher glycerol permeation, but performs at the same level as AQP7-WT. Likewise, the two individual GlpF-like mutations to the SF of AQP7 do not significantly change glycerol uptake vs. AQP7-WT. However, the AQP7-F74W&Y223F double mutation lowers glycerol uptake to be comparable with that of GlpF-WT (Figure 6C, blue and green bars).

^3^H-glycerol uptake also is significantly greater in GlpF-WT oocytes than in H_2_O-injected controls. It is noteworthy that mutating GlpF residues to their AQP7 counterparts did not increase glycerol uptake compared to GlpF-WT for any mutants (Figure 6C). Although the differences in ^3^H-glycerol uptake between the GlpF mutants and GlpF-WT generally do not reach statistical significance at the 0.05 level, the uptake values for the mutants are all indistinguishable from zero. Thus, it appears that the mutations to the EV or to the SF of GlpF blocks glycerol traffic.

#### Effect of Mutations on Rates of Cell Swelling in Normo-Osmotic Solutions Containing Glycerol

**FIGURE 7:**
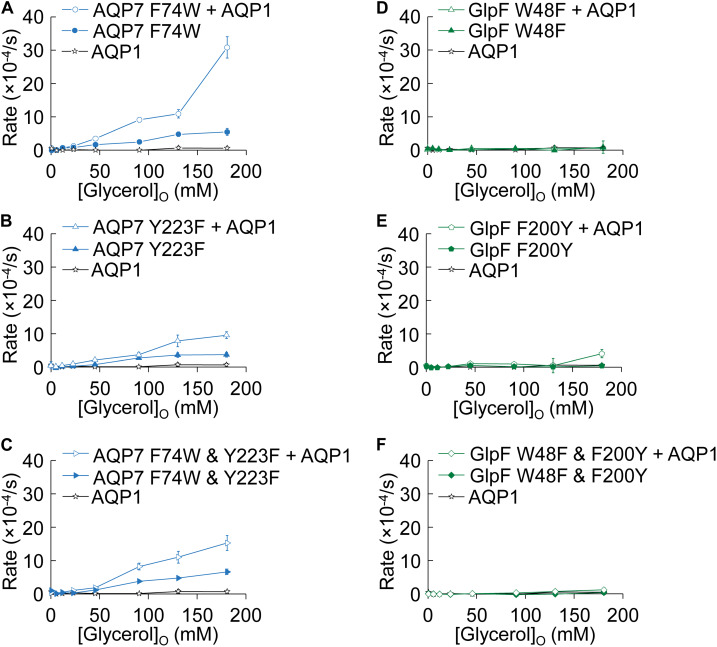
Normo-osmotic swelling of AQP7 and GlpF (± AQP1) expressing oocytes. **(A–C)** Oocyte swelling rates for AQP7 mutant channels expressed alone or co-expressed with AQP1 as [glycerol]_o_ in increased in a normo-osmotic buffer. **(D–F)** Oocyte swelling rates for GlpF mutant channels expressed alone or co-expressed with AQP1 as [glycerol]_o_ in increased in a normo-osmotic buffer. All data points represent mean ± S. E. M. These data and the number of oocytes and oocyte preparations/cRNA injections used to generate each point are detailed in Supplementary Tables S3, S4.

Figure 7 summarizes the results of experiments like those in Figure 2, in which we used osmotic swelling to assess glycerol uptake. Figures 7A–C summarize the effects of mutating AQP7 SF residues to their GlpF counterparts. AQP7-F74W ± AQP1 (Figure 7A) yields profiles not substantially different from AQP7-WT ± AQP1 (Figure 2A). Although AQP7-Y223F alone has a profile similar to that of AQP7-WT alone, co-expression with AQP1 does little to steepen the profile, particularly at high [glycerol]_o_ (Figure 7B). The AQP7-F74W&Y223F double mutant behaves similarly to the AQP7-Y223F single mutant (Figure 7C). Thus, in the presence of AQP1, glycerol fluxes via AQP7 constructs carrying the Y223F mutation saturate at substantially lower [glycerol]_o_ than other AQP7 constructs, consistent with the notion that Y223F (but not F74W) limits high (but has little effect on low) glycerol fluxes.

Figures 7D–F summarize comparable data on WT and mutated GlpF. We find that mutations to the SF of GlpF, alone (Figures 7D,E) or in combination (Figure 7F), abolish glycerol permeability, in keeping with the marked decreases in ^3^H-glycerol uptake shown in Figure 6C.

## Discussion

In humans, AQP7 mRNA levels are by far highest in adipose tissue, followed distantly by heart, small intestine, and kidney (see human tissue atlas^1^). During an overnight fast, the fall in plasma-insulin levels causes adipocytes in white adipose tissue (WAT) to convert triacylglycerols (TAGs) to fatty acids (FAs) and glycerol. Both products enter the circulation, FAs to provide energy to striated muscle and liver, and glycerol for gluconeogenesis, primarily in liver. Although AQP3, 9, 10, and 11 are also in some adipocytes (Madeira et al., 2015; Gotfryd et al., 2018), AQP7 is the dominant glycerol-efflux pathway from human WAT adipocytes (Kishida et al., 2000; Miranda et al., 2010; Laforenza et al., 2013; Iena and Lebeck, 2018), and presumably also plays a role in WAT water homeostasis.

In addition to its role in mediating glycerol efflux from adipocytes, AQP7 plays important roles in mediating glycerol uptake into mammalian cells, important examples being the uptake of this nutrient from the lumen of the small intestine (Laforenza et al., 2005, 2010, 2016; Ricanek et al., 2015), and glycerol reclamation from the lumen of renal proximal tubules (Sohara et al., 2005, 2009). Thus, our work has important implications for glycerol metabolism in health and disease (Lebeck, 2014).

### Key Results

1. Physiological experiments show that (a) AQP7 is better than GlpF as a glycerol channel, and (b) far better as a H_2_O channel. Thus, the permeability properties of AQP7 are similar to those of the sole aquaporin—an aquaglyceroporin—of *Plasmodium falciparum* (PfAQP; see Hansen et al., 2002; Aponte-Santamaría et al., 2010), a malarial parasite responsible for over 1 million human deaths annually.
2. The crystal structure of AQP7 reveals (a) the arrangement of the NAA (94–96) and NPS (226–228) motifs that form the NPA constriction in AQP7, where the van der Waals packing of A95 and P227 in AQP7 replaces that of the conserved prolines in GlpF; and (b) that residues F74 and Y223 in the SF establish a wedge that excludes glycerol, suggesting that our structure captures a closed state.
3. MD simulations demonstrate that (a) AQP7 is generally more flexible than GlpF; (b) AQP7-F74 in the SF undergoes rapid conformational changes from a closed to an open state, allowing glycerol to move through; (c) whereas in AQP7 glycerol passes through the SF region with no strong orientational confinement, in the more rigid GlpF, glycerol must adopt a parallel orientation along the pore axis; and (d) GlpF-W48 (position equivalent to AQP7-F74) exhibits rare but long-lasting conformational changes that prevent the passage of either glycerol or H_2_O.

### Structural Analysis

We present the structure (6N1G, deposited to the PDB on Nov 8, 2018) of the full-length AQP7 (the physiological condition), crystallized in the nominal absence of glycerol. This structure is resolved to 3.95 Å but the high crystal symmetry (F4_1_32) and additional presence of non-crystallographic symmetry (NCS) makes it possible, in symmetry-averaged maps, to resolve electron-density features of many amino-acid side-chains to near-atomic detail (Figure 3). The well resolved SF region reveals the closed state of the pore. Moreover, our structure shows clear average densities consistent with glycerol in all 4 monomeric pores of AQP7. The two NCS-related AQP7 dimers comprise two monomers with 3 glycerol molecules, and two monomers with 2 glycerol molecules in the monomeric pore.

A 3.7 Å resolution structure (6KXW, deposited to the PDB on Sept 13, 2019) of human AQP7—and reported in preliminary form (Zhang et al., 2019)—differs from ours in several respects:

1. The protein was trypsin-digested and thus truncated.
2. The crystal (P 21 21 21) has a lower symmetry.
3. The structure shows only chains A and C at 3.7 Å resolution, and many electron density features are generally less defined. For example, in the SF region, chains B and D exhibit no density for F74 and R229, which do not appear in the model.
4. For chains A and C, where SF residue side chain densities are present, an overlay with our structure shows that the 6KXW residue orientations are more in line with an ‘open state’ configuration, and are clearly different from the SF residues in our 6N1G structure.
5. Only two glycerol molecules are resolved in the 6KXW structure, one each in chains A and C. Neither of these glycerols in 6KXW are resolved in the same locations as any of the ten in our 6N1G structure.

A comparison of the de Maré et al. (2019) 1.9 Å 6QZI structure or the 2.2 Å 6QZJ structure (both of which were deposited to the PDB on March 11, 2019) vs. our 6N1G structure reveals that the two higher-resolution structures and ours are in general agreement for most of the protein. However, we note several important differences:

1. Closed vs. open state: Our 6N1G structure captures the ‘closed state’ of the SF region for glycerol permeability. We do not see a glycerol in the SF region, as is the case for both the GlpF and the two high-resolution AQP7 structures. The lack of a glycerol in our 6N1G structure does not reflect a lack of high-resolution data, but rather ‘steric restriction.’ Supplementary Figure S10 shows that all three SF residues in our closed-state structure exhibit different orientations from the same three residues in the open-state 1.9 Å 6QZI structure. Most notable is the downward rotamer observed for F74 in our closed-state structure vs. the upward F74 rotamer in the open-state structure.
2. In each of the two NCS-related AQP7 dimers in our 6N1G structure, one monomer has three glycerol molecules and the other has two glycerol molecules in its pore. In the 1.9 Å 6QZI structure, 5 glycerol molecules are modeled in the monomeric pore, whereas in the 2.2 Å 6QZJ structure, 4 glycerol molecules are present. We offer three possible explanations for the additional glycerol molecules in the two higher-resolution structures: (a) The higher resolution makes it more likely to identify glycerol molecules. (b) In addition to the glycerol present in the purification buffers, the higher-resolution crystals were soaked in 20% glycerol (1.9 Å 6QZI structure) or 5% glycerol (2.2 Å 6QZJ structure), enabling lower affinity sites to be fully occupied by glycerol. And (c) the open-state structures of 6QZI and 6QZJ increase the likelihood of glycerol entry into the monomeric pore. It is important to note that we crystalized AQP7 in the absence of extraneous glycerol and that no glycerol was employed during crystal freezing or data collection.
3. The protein expressed by de Maré et al. (2019) to obtain their 6QZI and 6QZJ structures had its N- and C-termini truncated. AQP7 lacking its termini is not physiologically appropriate because it fails to traffic appropriately to the plasma membrane (unpublished). However, while we expressed full-length protein to generate our 6N1G structure, we did not sufficiently resolve ordered densities for the N- or C-termini in order to build these regions.

Compared to both the 1.9 Å 6QZI and the 2.2 Å 6QZJ structures, two aspects of our presentation of the 6N1G structure are unique:

1. Y67 adopts two distinct positions (inward and outward) in each of the independent monomers in our structure (Figure 4A). Chains A and B, with inward-facing Y67 residues, contain two glycerol molecules, whereas chains C and D, with outward-facing Y67 residues, contain three glycerol molecules. In both high-resolution structures, Y67 adopts yet a different orientation (oxygen pointing toward G218).
2. In contrast to our structure, the pore-lining M58 sidechain in the high-resolution structures points into the pore (Figure 4A).

A rare set of exceptions to the highly conserved NPA/NPA motifs of AQPs occurs in the aquaglyceroporins of *P. falciparum* and several other *Plasmodium* species, where a pair of complementary amino-acid replacements produce NLA/NPS. AQP7 is another exception. In our structure, the interaction area of the NAA/NPS motif is ∼10% smaller than the interaction area of the conserved NPA/NPA motif of GlpF. Our data on the interaction area are also in agreement with those for the NAA/NPS motif in the 1.9 Å 6QZI structure (de Maré et al., 2019).

### MD Summary/Analysis

Our MD simulations show that, compared to GlpF, AQP7 (a) exhibits much larger variations in ΔG profiles of individual pores—revealing significantly lower barriers at the SF region (Figure 5A vs. Figure 5C)—and (b) is more flexible (Figure 5E). Specifically, MD simulations reveal large and frequent F74 movements (Figures 5F,G,J) that open and close the SF, which our crystal structure captures in a closed state (Figure 4C). These conformational changes allow glycerol—with almost no orientational constraints (Figure 5B)—to pass AQP7’s SF region. In contrast, the rigid SF of GlpF reveals few movements of W48 (Figure 5L) and forces the transiting glycerol molecule to assume a single orientation (Figure 5D; see Hénin et al., 2008). Consistent with this constraint is the presence of a glycerol molecule at the SF of the GlpF—but not the AQP7—crystal structure. The conformational flexibility of the SF in AQP7 may thus result in greater permeabilities to both glycerol and H_2_O, as we measured in our oocyte experiments (Figures 1, 2, 6, 7) accordingly, we observed three glycerol translocation events across the SF in AQP7, but a single event in GlpF during the 3 × 1-μs flooding simulations (Supplementary Figure S4).

A global comparison of the interaction network of GlpF and AQP7 using RING 2.0 (Piovesan et al., 2016) may provide useful qualitative information: The sum of all non-covalent interaction energies is 1,733 kcal/mol in GlpF, but only 1,372 kcal/mol in AQP7, with average energies per residue of 6.81 kcal/mol in GlpF, but only 5.5 kcal/mol in AQP7—differences consistent with the higher conformational flexibility of AQP7 compared to GlpF.

Although the above observations—centered on the greater flexibility of AQP7 vs. GlpF—are suggestive of a higher glycerol permeability in AQP7, we note that glycerol passage through the monomeric pores is a slow event on the atomistic-MD timescale. Thus, despite the extended length of the present simulations, we cannot collect sufficient statistics from these equilibrium simulations to make a quantitative comparison of glycerol permeability in the two aquaglyceroporins. Nevertheless, the rare but long-lasting conformational changes of GlpF-W48 that block the pore may also contribute to the lower glycerol and water permeabilities of GlpF, compared to AQP7, found in our oocyte swelling experiments.

A comparison of the MD analyses of de Maré et al. (2019) and those in the present paper reveals several similarities:

1. The extended electron densities in the two structures by de Maré et al. indicate that the glycerol molecule rotates—especially in the NPA—as it permeates the pore. Consistent with this notion, our MD analysis shows that glycerol is subject to no orientational constraints as it passes through the SF and along the rest of the channel (Figure 5B).
2. The studies of both de Maré et al. and this study show that glycerol occupancy in the pore reduces water permeability.
3. The PMF profiles for glycerol in both studies identify two barriers along the pathway, the first located at the SF, and the second located below the intracellular half of the NPA.

A comparison of two sets of MD analyses reveals several differences.

• Whereas de Maré et al. initiated their MD simulations with the SF of their AQP7 in the “open-state,” we initiated MD simulations with the SF of our AQP7 structure in the “closed state.”
• Whereas at *t* = 0, the flooding simulations of de Maré et al. started with 16 glycerol molecules in the bulk solution, we started with 200.
• Whereas de Maré et al. performed simulations of water permeation with four X-ray bound glycerols in the pore, we restrained a single glycerol at the NPA region.

Finally, our MD analyses lead to observations that are unique to the present study:

1. We performed MD simulations on AQP1 (a classical aquaporin), AQP7 (an aquaglyceroporin), and GlpF (a glyceroporin with limited water permeability).
2. We performed structural dynamics analyses of the AQP7 and glycerol molecule that revealed:
  a. The AQP7 backbone is much more flexible than GlpF (Figure 5E).
  b. Glycerol orientation is unrestrained as it transitions through the AQP7 monomeric pore while it is tightly constrained in GlpF (Figures 5B,D).
3. Our MD simulations reveal that F74 can alternate rapidly between “down” (as observed our 6N1G structure) and “up” conformations (as observed in 6QZI structures), and that these conformational changes are important in glycerol transition through the SF (Figure 5 and Supplementary Figure S9).

### Physiology Summary/Analysis

The most critical insight into the interaction of glycerol and H_2_O fluxes comes from experiments in which we monitored oocyte swelling/shrinking under different conditions (Figures 1, 2). AQP7-expressing oocytes swell moderately when exposed to hypo-osmotic solutions (Figure 2A, filled blue diamonds), consistent with their significant *P*_f_ (Figure 1A, filled blue and blue-edged white bars) and ^3^H-glycerol uptake (Figure 6C, blue bars). GlpF-expressing oocytes do not swell at all (Figure 2B, green filled squares), confirming early oocyte experiments (Ishibashi et al., 1997). When we subject AQP7-expressing oocytes to a hyper-osmolal NaCl solution (Figure 1B, blue-rimmed white bar), we observe a shrinkage rate similar to that of AQP1-expressing oocytes (Figure 1B, black-rimmed white bar), as expected from *P*_f_ measurements (Figures 1A, 6A). Surprisingly, AQP7-expressing oocytes swell (rather than shrink) at high rate in a hyper-osmolal glycerol solution (Figure 1B, striped blue bar). Thus, the glycerol and H_2_O permeabilities of AQP7—but not GlpF—must be so high that the glycerol influx rapidly reverses the osmotic gradient in the extra-/intracellular nanodomains around AQP7, drawing H_2_O into the cell via the same monomeric AQP7 pores.

Co-expression with AQP1 dramatically enhances swelling, with the effect being about twice as great in AQP7 + AQP1 vs. GlpF + AQP1 oocytes (Figure 2, open symbols). The data of Figures 1, 2 how that, on a macroscopic time scale, AQP7 conducts large amounts of glycerol and H_2_O, whereas GlpF conducts smaller amounts glycerol, which nonetheless severely throttles H_2_O permeation. We speculate that AQP7 is also permeable to the polyol mannitol, the uptake of which (along with osmotically obligated H_2_O) would explain the smaller negative swelling rate in ND96 + Mannitol vs. NaCl (Figure 1B, blue solid bar vs. adjacent blue-rimmed white bar).

Investigators have long been intrigued by the low *P*_f_ of GlpF, and speculated on a role of glycerol passage (de Groot and Grubmüller, 2001). MD simulations provided the first mechanistic details of glycerol throttling in GlpF (Chen, 2013), predicting a 7.3 × reduction of *P*_f_ in the presence of 14 mM glycerol. Our 200/250 ns MD simulations of *apo* and glycerol-bound states yield equal *P*_*d*_ values of the apo states of GlpF and AQP7 and, for both, 80 × reductions in ∼500 mM glycerol (Supplementary Figure S6). Thus, MD simulations predict that when glycerol is present in the pore, it obstructs H_2_O permeation. For ion channels, open probability (*P*_o_) describes the fraction of time that the channel conducts. For H_2_O conduction by aquaglyceroporins, occupancy by glycerol is analogous to (1 – *P*_o_). Regrettably, even our 3 × 1-μs flooding simulations (Supplementary Figure S4) are far too brief to provide statistically meaningful insight into glycerol occupancy, arguing for greatly extended MD simulations.

In oocyte assays, we also examined the effects of swapping residues at equivalent SF positions between AQP7 (F74 and Y223) and GlpF (W48 and F200). AQP7 retains all/some function with single point mutations, possibly reflecting its flexibility. The opposite mutations leave GlpF nearly non-functional, possibly because the relatively stiff GlpF molecule cannot accommodate these mutations, resulting in a blocked channel.

Based on the present oocyte-swelling experiments (Figures 1A, 6A)—and previous AQP1 studies (Musa-Aziz et al., 2009; Geyer et al., 2013)—*P*_f_ is significantly higher in oocytes expressing AQP1 than AQP7. Our MD analyses, however, predict the opposite in the absence of glycerol (Supplementary Figure S6A). We propose that glycerol, present in the cytoplasm of virtually all cells, diffuses into the monomeric pores of AQP7, where its occupancy slows the movement of H_2_O. We cannot estimate glycerol occupancy of AQP7 because of the unavailability of [glycerol]_*i*_ data and the need to extend our MD simulations by orders of magnitude.

### Physiological Advantage of AQP7 (vs. GlpF) in Adipocytes

The functional advantage for mammals to utilize AQP7 (high H_2_O and glycerol permeability), rather than a more GlpF-like AQP, is clearest for WAT adipocytes during lipolysis. Epinephrine, acting via adenylyl cyclase (AC), leads to increased activity of adipose triglyceride lipase (ATGL) and hormone-sensitive lipase (HSL), and thus the hydrolysis of triacylglycerols (TAGs) to release 3 fatty acid (FA) molecules plus glycerol (Sztalryd et al., 2003; Miyoshi et al., 2006; Brasaemle, 2007; McDonough et al., 2013). Long-chain acyl-CoAs inhibit ATGL, and FAs inhibit AC and possibly also HSL (Fain and Shepherd, 1975; Jepson and Yeaman, 1992; Nagy et al., 2014). We propose that, early during lipolysis, the build-up of FAs and glycerol creates an osmotic gradient that leads to H_2_O influx via AQP7, thereby diluting FAs and reducing their inhibitory effects. In the lipolytic steady state, fatty-acid transport proteins (FATPs) and fatty acid translocase (FAT) export the FAs (Gimeno, 2007), and AQP7 exports glycerol. Finally, as lipolysis wanes, AQP7 additionally would mediate the efflux of osmotically obligated H_2_O until the adipocyte returns to its initial H_2_O-electrolyte status.

In addition to abundant mRNA encoding AQP7, human adipocytes have much lower levels of mRNA encoding AQP10 (Miranda et al., 2010; Laforenza et al., 2013). Intracellular pH (pH_*i*_) measurements of isolated human adipocytes show that isoproterenol not only stimulates lipolysis but also leads to a slow, small acidification (from pH_*i*_ ∼6.6 to ∼6.5 over 40 min), presumably reflecting the release of FAs from glycerol. Based on the recent 2.3 Å–resolution structure of human AQP10 as well as the associated MD analyses, Gotfryd et al. (2018) identified a histidine at position 80—located near the cytosolic mouth of the monomeric pore—as a potential pH_*i*_ sensor. They propose that when H80 is unprotonated, the pore is obstructed and that, in this conformation, H80 has a pKa of ∼3.6. A decrease in pH_*i*_ would protonate H80, leading to a series of conformational changes that open the pore and also increase the pKa to 7.1. In experiments on proteoliposomes containing reconstituted AQP10, they show that lowering the cytosol-side pH from 7.4 to 5.5 causes the AQP10-dependent glycerol (but not water) flux to rise. In contrast, this pH decrease blocks glycerol fluxes through AQPs 3, 7, and 9. Thus, the authors propose that this novel pH switch allows AQP10 to mediate glycerol fluxes at low pH_*i*_—induced by lipolysis—where other aquaglyceroporins would fail. However, the AQP10-mediated glycerol fluxes would become physiologically significant only at extremely low pH_*i*_ (∼5.5), far lower than the values observed in adipocytes undergoing lipolysis. Furthermore, for three reasons, one would expect the lipolysis-induced decreases in pH_*i*_ to be self-limited: (a) adipocytes export FAs via FATPs (Gimeno, 2007), minimizing FA deprotonation inside the adipocyte; (b) adipocytes possess potent pH_*i*_-regulatory systems (Gabrielsson et al., 2000; Chen and Mackintosh, 2009); and (c) because hormone-sensitive lipase has a pH maximum around pH 7.0 (Hollenberg et al., 1970), lipolysis-induced decreases in pH_*i*_ would tend to put a brake on lipolysis.

### Gas Permeability

Certain AQPs can conduct CO_2_ (Cooper and Boron, 1998; Nakhoul et al., 1998; Musa-Aziz et al., 2009; Itel et al., 2012; Geyer et al., 2013; Tsiavaliaris et al., 2015) or NH_3_ (Litman et al., 2009; Musa-Aziz et al., 2009; Geyer et al., 2013). AQP1 conducts both, and MD simulations suggest that CO_2_ could move through AQP1 via the relatively hydrophilic monomeric pores, as well as the hydrophobic central pore (Wang et al., 2007). Although mammalian aquaglyceroporins 3, 7 and 9 conduct NH_3_, only AQP9 conducts CO_2_ (Geyer et al., 2013). Lack of CO_2_ conduction through the AQP7 monomeric pores could reflect throttling by glycerol, derived from cellular metabolism.

## Conclusion

Our crystal structure, MD simulations, and physiological studies show that, in living cells, AQP7 is both a better H_2_O channel and a better glycerol facilitator than GlpF. These properties are likely related to the much greater conformational flexibility of AQP7, rare but persistent conformational transitions of GlpF-W48 that block both glycerol and H_2_O movements, and more effective throttling of H_2_O by resident glycerol in GlpF. In contrast, the rapid conformational shifts of the analogous AQP7-F74 could be a novel form of glycerol gating.

## Data Availability Statement

The datasets generated for this study can be found in the Protein Data Bank, www.pdb.org (PDB ID code 6N1G); deposited on March 11, 2019 and released on November 13, 2019.

## Ethics Statement

Protocols for housing and handling of *Xenopus laevis* were approved by the Institutional Animal Care and Use Committee at Case Western Reserve University.

## Author Contributions

AV-F performed the crystallization of AQP7, analyzed the data, and wrote the relevant results sections of the manuscript, and together with FM and PM, drafted the manuscript. DL along with AV-F performed X-ray data collection, structure determination and refinement. FM generated the AQP7 and GlpF constructs for oocyte studies; performed glycerol-uptake, osmotic-swelling, and surface-biotinylation assays; analyzed data and wrote the relevant results sections of the manuscript. PM performed MD simulations, analyzed data and wrote the relevant results sections of the manuscript. TK refined the structure, prepared coordinates for deposition, and deposited the 6N1G AQP7 structure to the PDB databank. ET, WB, and AE supervised the project. All authors contributed to the drafting of the discussion and conclusions. WB, FM, and AE edited the manuscript.

## Conflict of Interest

The authors declare that the research was conducted in the absence of any commercial or financial relationships that could be construed as a potential conflict of interest.

## Footnotes

1 https://www.proteinatlas.org/ENSG00000165269-AQP7/tissue

## References

[B1] Aponte-Santamaría C. Hub J. S. de Groot B. L. (2010). Dynamics and energetics of solute permeation through the *Plasmodium falciparum* aquaglyceroporin. *Phys. Chem. Chem. Phys.* 12 10246–10254. 10.1039/c004384m 20607193

[B2] Bienert G. P. Chaumont F. (2014). Aquaporin-facilitated transmembrane diffusion of hydrogen peroxide. *Biochim. Biophys. Acta* 1840 1596–1604. 10.1016/j.bbagen.2013.09.017 24060746

[B3] Borgnia M. Nielsen S. Engel A. Agre P. (1999). Cellular and molecular biology of the aquaporin water channels. *Annu. Rev. Biochem.* 68 425–458. 10.1146/annurev.biochem.68.1.425 10872456

[B4] Borgnia M. J. Agre P. (2001). Reconstitution and functional comparison of purified GlpF and AqpZ, the glycerol and water channels from *Escherichia coli*. *Proc. Natl. Acad. Sci. U.S.A.* 98 2888–2893. 10.1073/pnas.051628098 11226336 PMC30235

[B5] Brasaemle D. L. (2007). Thematic review series: adipocyte biology. The perilipin family of structural lipid droplet proteins: stabilization of lipid droplets and control of lipolysis. *J. Lipid Res.* 48 2547–2559. 10.1194/jlr.R700014-JLR200 17878492

[B6] Chen L. Y. (2013). Glycerol modulates water permeation through *Escherichia coli* aquaglyceroporin GlpF. *Biochim. Biophys. Acta* 1828 1786–1793. 10.1016/j.bbamem.2013.03.008 23506682 PMC3761968

[B7] Chen S. Mackintosh C. (2009). Differential regulation of NHE1 phosphorylation and glucose uptake by inhibitors of the ERK pathway and p90RSK in 3T3-L1 adipocytes. *Cell. Signal.* 21 1984–1993. 10.1016/j.cellsig.2009.09.009 19765648

[B8] Cooper G. J. Boron W. F. (1998). Effect of pCMBS on CO_2_ permeability of *Xenopus* oocytes expressing aquaporin 1 or its C189S mutant. *Am. J. Physiol.* 275 C1481–C1486. 9843709 10.1152/ajpcell.1998.275.6.C1481

[B9] de Groot B. L. Grubmüller H. (2001). Water permeation across biological membranes: mechanism and dynamics of aquaporin-1 and GlpF. *Science* 294 2353–2357. 10.1126/science.1062459 11743202

[B10] de Maré S. W. Venskutonytë R. Eltschkner S. de Groot B. L. Lindkvist-Petersson K. (2019). Structural basis for glycerol efflux and selectivity of human aquaporin 7. *Structure* 28 215.e3–222.e3. 10.1016/j.str.2019.11.011 31831212

[B11] Fain J. N. Shepherd R. E. (1975). Free fatty acids as feedback regulators of adenylate cyclase and cyclic 3′:5′-AMP accumulation in rat fat cells. *J. Biol. Chem.* 250 6586–6592. 169252

[B12] Fu D. Libson A. Miercke L. J. Weitzman C. Nollert P. Krucinski J. (2000). Structure of a glycerol-conducting channel and the basis for its selectivity. *Science* 290 481–486. 11039922 10.1126/science.290.5491.481

[B13] Gabrielsson B. L. Carlsson B. Carlsson L. M. (2000). Partial genome scale analysis of gene expression in human adipose tissue using DNA array. *Obes. Res.* 8 374–384. 10.1038/oby.2000.45 10968729

[B14] Geyer R. R. Musa-Aziz R. Qin X. Boron W. F. (2013). Relative CO_2_/NH_3_ selectivities of mammalian aquaporins 0-9. *Am. J. Physiol., Cell Physiol.* 304 C985–C994. 10.1152/ajpcell.00033.2013 23485707

[B15] Gimeno R. E. (2007). Fatty acid transport proteins. *Curr. Opin. Lipidol.* 18 271–276. 10.1097/MOL.0b013e3281338558 17495600

[B16] Gonen T. Walz T. (2006). The structure of aquaporins. *Q. Rev. Biophys.* 39 361–396. 10.1017/S0033583506004458 17156589

[B17] Gotfryd K. Mósca A. F. Missel J. W. Truelsen S. F. Wang K. Spulber M. (2018). Human adipose glycerol flux is regulated by a pH gate in AQP10. *Nat. Commun.* 9:4749. 10.1038/s41467-018-07176-z 30420639 PMC6232157

[B18] Hansen M. Kun J. F. J. Schultz J. E. Beitz E. (2002). A single, bi-functional aquaglyceroporin in blood-stage *Plasmodium falciparum* malaria parasites. *J. Biol. Chem.* 277 4874–4882. 10.1074/jbc.M110683200 11729204

[B19] Hara-Chikuma M. Sohara E. Rai T. Ikawa M. Okabe M. Sasaki S. (2005). Progressive adipocyte hypertrophy in aquaporin-7-deficient mice: adipocyte glycerol permeability as a novel regulator of fat accumulation. *J. Biol. Chem.* 280 15493–15496. 10.1074/jbc.C500028200 15746100

[B20] Hénin J. Tajkhorshid E. Schulten K. Chipot C. (2008). Diffusion of glycerol through *Escherichia coli* aquaglyceroporin GlpF. *Biophys. J.* 94 832–839. 10.1529/biophysj.107.115105 17921212 PMC2186255

[B21] Hermo L. Schellenberg M. Liu L. Y. Dayanandan B. Zhang T. Mandato C. A. (2008). Membrane domain specificity in the spatial distribution of aquaporins 5, 7, 9, and 11 in efferent ducts and epididymis of rats. *J. Histochem. Cytochem.* 56 1121–1135. 10.1369/jhc.2008.951947 18796408 PMC2583908

[B22] Heymann J. B. Engel A. (1999). Aquaporins: phylogeny. Structure, and physiology of water channels. *News Physiol. Sci.* 14 187–193. 11390849 10.1152/physiologyonline.1999.14.5.187

[B23] Hibuse T. Maeda N. Funahashi T. Yamamoto K. Nagasawa A. Mizunoya W. (2005). Aquaporin 7 deficiency is associated with development of obesity through activation of adipose glycerol kinase. *Proc. Natl. Acad. Sci. U.S.A.* 102 10993–10998. 10.1073/pnas.0503291102 16009937 PMC1182435

[B24] Hollenberg C. H. Vost A. Patten R. L. (1970). “Regulation of Adipose Mass: Control of Fat Cell Development and Lipid Content,” in *Proceedings of the 1969 Laurentian Hormone Conference Recent Progress in Hormone Research*, ed. Astwood E. B. Boston: Academic Press, 463–503. 10.1016/b978-0-12-571126-5.50015-7 4319351

[B25] Holm S. (1979). A simple sequentially rejective multiple test procedure. *Scand. J. Stat.* 6 65–70.

[B26] Hub J. S. de Groot B. L. (2008). Mechanism of selectivity in aquaporins and aquaglyceroporins. *Proc. Natl. Acad. Sci. U.S.A.* 105 1198–1203. 10.1073/pnas.0707662104 18202181 PMC2234115

[B27] Iena F. M. Lebeck J. (2018). Implications of aquaglyceroporin 7 in energy metabolism. *Int. J. Mol. Sci.* 19:154. 10.3390/ijms19010154 29300344 PMC5796103

[B28] Ishibashi K. Imai M. Sasaki S. (2000). Cellular localization of aquaporin 7 in the rat kidney. *Exp. Nephrol.* 8 252–257. 10940724 10.1159/000020676

[B29] Ishibashi K. Kuwahara M. Gu Y. Kageyama Y. Tohsaka A. Suzuki F. (1997). Cloning and functional expression of a new water channel abundantly expressed in the testis permeable to water, glycerol, and urea. *J. Biol. Chem.* 272 20782–20786. 9252401 10.1074/jbc.272.33.20782

[B30] Itel F. Al-Samir S. Öberg F. Chami M. Kumar M. Supuran C. T. (2012). CO_2_ permeability of cell membranes is regulated by membrane cholesterol and protein gas channels. *FASEB J.* 26 5182–5191. 10.1096/fj.12-209916 22964306

[B31] Jepson C. A. Yeaman S. J. (1992). Inhibition of hormone-sensitive lipase by intermediary lipid metabolites. *FEBS Lett.* 310 197–200. 1356829 10.1016/0014-5793(92)81328-j

[B32] Kishida K. Kuriyama H. Funahashi T. Shimomura I. Kihara S. Ouchi N. (2000). Aquaporin adipose, a putative glycerol channel in adipocytes. *J. Biol. Chem.* 275 20896–20902. 10.1074/jbc.M001119200 10777495

[B33] Laforenza U. Bottino C. Gastaldi G. (2016). Mammalian aquaglyceroporin function in metabolism. *Biochim. Biophys. Acta Biomembr.* 1858 1–11. 10.1016/j.bbamem.2015.10.004 26456554

[B34] Laforenza U. Gastaldi G. Grazioli M. Cova E. Tritto S. Faelli A. (2005). Expression and immunolocalization of aquaporin-7 in rat gastrointestinal tract. *Biol. Cell* 97 605–613. 10.1042/BC20040090 15943587

[B35] Laforenza U. Miceli E. Gastaldi G. Scaffino M. F. Ventura U. Fontana J. M. (2010). Solute transporters and aquaporins are impaired in celiac disease. *Biol. Cell* 102 457–467. 10.1042/BC20100023 20415666

[B36] Laforenza U. Scaffino M. F. Gastaldi G. (2013). Aquaporin-10 represents an alternative pathway for glycerol efflux from human adipocytes. *PLoS One* 8:e54474. 10.1371/journal.pone.0054474 23382902 PMC3558521

[B37] Lebeck J. (2014). Metabolic impact of the glycerol channels AQP7 and AQP9 in adipose tissue and liver. *J. Mol. Endocrinol.* 52 R165–R178. 10.1530/JME-13-0268 24463099

[B38] Litman T. Søgaard R. Zeuthen T. (2009). Ammonia and urea permeability of mammalian aquaporins. *Handb. Exp. Pharmacol.* 19 327–358. 10.1007/978-3-540-79885-9_17 19096786

[B39] Liu Z. Shen J. Carbrey J. M. Mukhopadhyay R. Agre P. Rosen B. P. (2002). Arsenite transport by mammalian aquaglyceroporins AQP7 and AQP9. *Proc. Natl. Acad. Sci. U.S.A.* 99 6053–6058. 10.1073/pnas.092131899 11972053 PMC122900

[B40] Madeira A. Moura T. F. Soveral G. (2015). Aquaglyceroporins: implications in adipose biology and obesity. *Cell. Mol. Life Sci.* 72 759–771. 10.1007/s00018-014-1773-1772 25359234 PMC11113391

[B41] McDonough P. M. Maciejewski-Lenoir D. Hartig S. M. Hanna R. A. Whittaker R. Heisel A. (2013). Differential phosphorylation of perilipin 1A at the initiation of lipolysis revealed by novel monoclonal antibodies and high content analysis. *PLoS One* 8:e55511. 10.1371/journal.pone.0055511 23405163 PMC3566132

[B42] Mehanna E. T. Barakat B. M. ElSayed M. H. Tawfik M. K. (2018). An optimized dose of raspberry ketones controls hyperlipidemia and insulin resistance in male obese rats: effect on adipose tissue expression of adipocytokines and Aquaporin 7. *Eur. J. Pharmacol* 832 81–89. 10.1016/j.ejphar.2018.05.028 29787773

[B43] Miranda M. Escoté X. Ceperuelo-Mallafré V. Alcaide M. J. Simón I. Vilarrasa N. (2010). Paired subcutaneous and visceral adipose tissue aquaporin-7 expression in human obesity and type 2 diabetes: differences and similarities between depots. *J. Clin. Endocrinol. Metab.* 95 3470–3479. 10.1210/jc.2009-2655 20463097

[B44] Miyoshi H. Souza S. C. Zhang H.-H. Strissel K. J. Christoffolete M. A. Kovsan J. (2006). Perilipin promotes hormone-sensitive lipase-mediated adipocyte lipolysis via phosphorylation-dependent and -independent mechanisms. *J. Biol. Chem.* 281 15837–15844. 10.1074/jbc.M601097200 16595669

[B45] Miyoshi T. Yamaguchi T. Ogita K. Tanaka Y. Ishibashi K.-I. Ito H. (2017). Quantitative analysis of aquaporin expression levels during the development and maturation of the inner ear. *J. Assoc. Res. Otolaryngol.* 18 247–261. 10.1007/s10162-016-0607-603 28004290 PMC5352615

[B46] Murata K. Mitsuoka K. Hirai T. Walz T. Agre P. Heymann J. B. (2000). Structural determinants of water permeation through aquaporin-1. *Nature* 407 599–605. 10.1038/35036519 11034202

[B47] Musa-Aziz R. Boron W. F. Parker M. D. (2010). Using fluorometry and ion-sensitive microelectrodes to study the functional expression of heterologously-expressed ion channels and transporters in *Xenopus* oocytes. *Methods* 51 134–145. 10.1016/j.ymeth.2009.12.012 20051266 PMC2905798

[B48] Musa-Aziz R. Chen L.-M. Pelletier M. F. Boron W. F. (2009). Relative CO_2_/NH_3_ selectivities of AQP1, AQP4, AQP5, AmtB, and RhAG. *Proc. Natl. Acad. Sci. U.S.A.* 106 5406–5411. 10.1073/pnas.0813231106 19273840 PMC2664022

[B49] Nagy H. M. Paar M. Heier C. Moustafa T. Hofer P. Haemmerle G. (2014). Adipose triglyceride lipase activity is inhibited by long-chain acyl-coenzyme A. *Biochim. Biophys. Acta* 1841 588–594. 10.1016/j.bbalip.2014.01.005 24440819 PMC3988850

[B50] Nakhoul N. L. Davis B. A. Romero M. F. Boron W. F. (1998). Effect of expressing the water channel aquaporin-1 on the CO_2_ permeability of *Xenopus* oocytes. *Am. J. Physiol.* 274 C543–C548. 9486145 10.1152/ajpcell.1998.274.2.C543

[B51] Nejsum L. N. Elkjaer M. Hager H. Frokiaer J. Kwon T. H. Nielsen S. (2000). Localization of aquaporin-7 in rat and mouse kidney using RT-PCR, immunoblotting, and immunocytochemistry. *Biochem. Biophys. Res. Commun.* 277 164–170. 10.1006/bbrc.2000.3638 11027658

[B52] Nielsen S. Frøkiaer J. Marples D. Kwon T.-H. Agre P. Knepper M. A. (2002). Aquaporins in the kidney: from molecules to medicine. *Physiol. Rev.* 82 205–244. 10.1152/physrev.00024.2001 11773613

[B53] Piovesan D. Minervini G. Tosatto S. C. E. (2016). The RING 2.0 web server for high quality residue interaction networks. *Nucleic Acids Res.* 44 W367–W374. 10.1093/nar/gkw315 27198219 PMC4987896

[B54] Ricanek P. Lunde L. K. Frye S. A. Støen M. Nygård S. Morth J. P. (2015). Reduced expression of aquaporins in human intestinal mucosa in early stage inflammatory bowel disease. *Clin. Exp. Gastroenterol.* 8 49–67. 10.2147/CEG.S70119 25624769 PMC4296881

[B55] Rodríguez A. Catalán V. Gómez-Ambrosi J. Frühbeck G. (2006). Role of aquaporin-7 in the pathophysiological control of fat accumulation in mice. *FEBS Lett.* 580 4771–4776. 10.1016/j.febslet.2006.07.080 16919625

[B56] Saito K. Kageyama Y. Okada Y. Kawakami S. Kihara K. Ishibashi K. (2004). Localization of aquaporin-7 in human testis and ejaculated sperm: possible involvement in maintenance of sperm quality. *J. Urol.* 172 2073–2076. 15540792 10.1097/01.ju.0000141499.08650.ab

[B57] Skowronski M. T. Lebeck J. Rojek A. Praetorius J. Füchtbauer E.-M. Frøkiaer J. (2007). AQP7 is localized in capillaries of adipose tissue, cardiac and striated muscle: implications in glycerol metabolism. *Am. J. Physiol. Renal Physiol.* 292 F956–F965. 10.1152/ajprenal.00314.2006 17077387

[B58] Sohara E. Rai T. Miyazaki J. Verkman A. S. Sasaki S. Uchida S. (2005). Defective water and glycerol transport in the proximal tubules of AQP7 knockout mice. *Am. J. Physiol. Renal Physiol.* 289 F1195–F1200. 10.1152/ajprenal.00133.2005 15998844

[B59] Sohara E. Rai T. Sasaki S. Uchida S. (2006). Physiological roles of AQP7 in the kidney: lessons from AQP7 knockout mice. *Biochim. Biophys. Acta* 1758 1106–1110. 10.1016/j.bbamem.2006.04.002 16860289

[B60] Sohara E. Uchida S. Sasaki S. (2009). Function of aquaporin-7 in the kidney and the male reproductive system. *Handb. Exp. Pharmacol.* 19 219–231. 10.1007/978-3-540-79885-9_11 19096780

[B61] Sui H. Han B. G. Lee J. K. Walian P. Jap B. K. (2001). Structural basis of water-specific transport through the AQP1 water channel. *Nature* 414 872–878. 10.1038/414872a 11780053

[B62] Sztalryd C. Xu G. Dorward H. Tansey J. T. Contreras J. A. Kimmel A. R. (2003). Perilipin A is essential for the translocation of hormone-sensitive lipase during lipolytic activation. *J. Cell Biol.* 161 1093–1103. 10.1083/jcb.200210169 12810697 PMC2172984

[B63] Tsiavaliaris G. Itel F. Hedfalk K. Al-Samir S. Meier W. Gros G. (2015). Low CO_2_ permeability of cholesterol-containing liposomes detected by stopped-flow fluorescence spectroscopy. *FASEB J.* 29 1780–1793. 10.1096/fj.14-263988 25609423

[B64] Verkman A. S. (2012). Aquaporins in clinical medicine. *Annu. Rev. Med.* 63 303–316. 10.1146/annurev-med-043010-193843 22248325 PMC3319404

[B65] Wang Y. Cohen J. Boron W. F. Schulten K. Tajkhorshid E. (2007). Exploring gas permeability of cellular membranes and membrane channels with molecular dynamics. *J. Struct. Biol.* 157 534–544. 10.1016/j.jsb.2006.11.008 17306562

[B66] Zhang L. Yao D. Zhou F. Zhang Q. Xia Y. Wang Q. (2019). The structural basis for glycerol permeation by human AQP7. *bioRxiv* [Preprint]. 10.1101/858332 36654284

[B67] Zhu C. Chen Z. Jiang Z. (2016). Expression, distribution and role of aquaporin water channels in human and animal stomach and intestines. *Int. J. Mol. Sci.* 17:1399. 10.3390/ijms17091399 27589719 PMC5037679

