# Supplementary material for: Aquaporin-7: A Dynamic Aquaglyceroporin With Greater Water and Glycerol Permeability Than Its Bacterial Homolog GlpF
Source: Front Physiol. 2020 Jun 30;11:728. doi: 10.3389/fphys.2020.00728 (PMC7339978; doi:10.3389/fphys.2020.00728)
Supplement: Supplementary file 1 [file Presentation_1.pdf]

Supplementary Figures

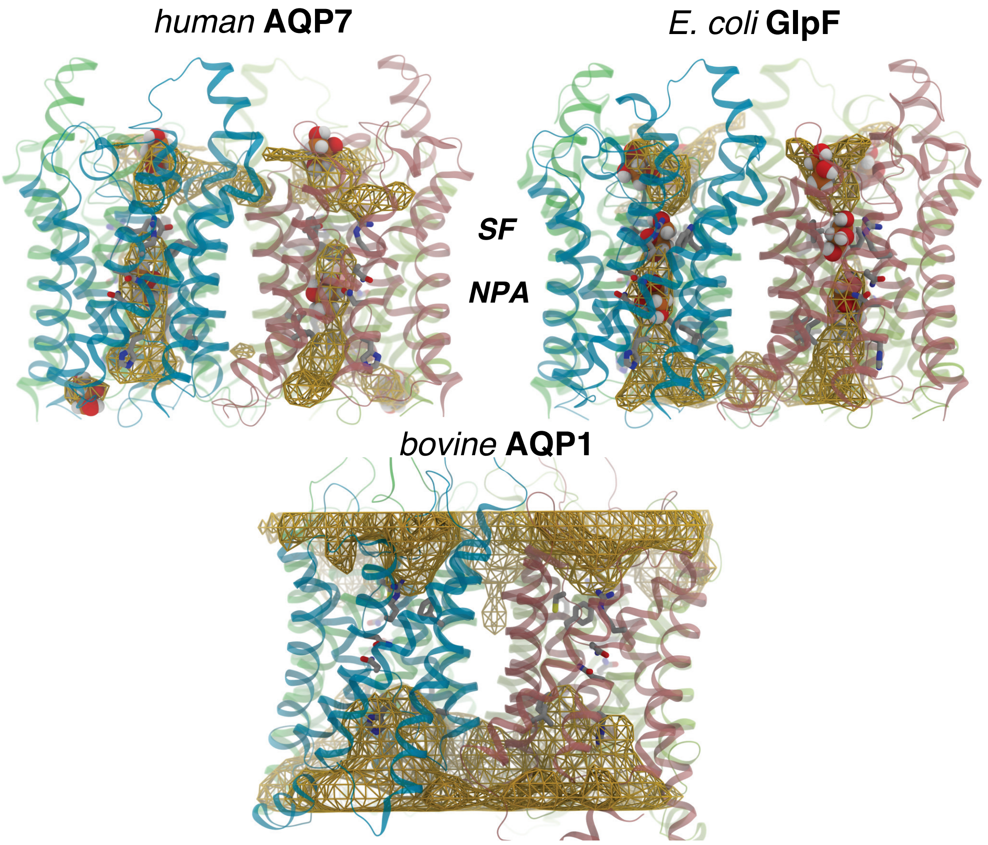

Supplementary Figure S1: Highly occupied sites of glycerol molecules in AQP7, GlpF and AQP1 observed during microsecond-long flooding simulations of 200 glycerol molecules. The orange transparent surfaces shown in the AQP7 and GlpF represent the apparent insertion probabilities of 5%; the one in the AQP1 represents the probability of 0.05%. We show the glycerol molecules in the crystal structures of AQP7 and GlpF in vdW. Glycerol molecules partitioned into the monomeric pores of AQP7 and GlpF. We observe no glycerol penetration in AQP1, which has narrower pores.

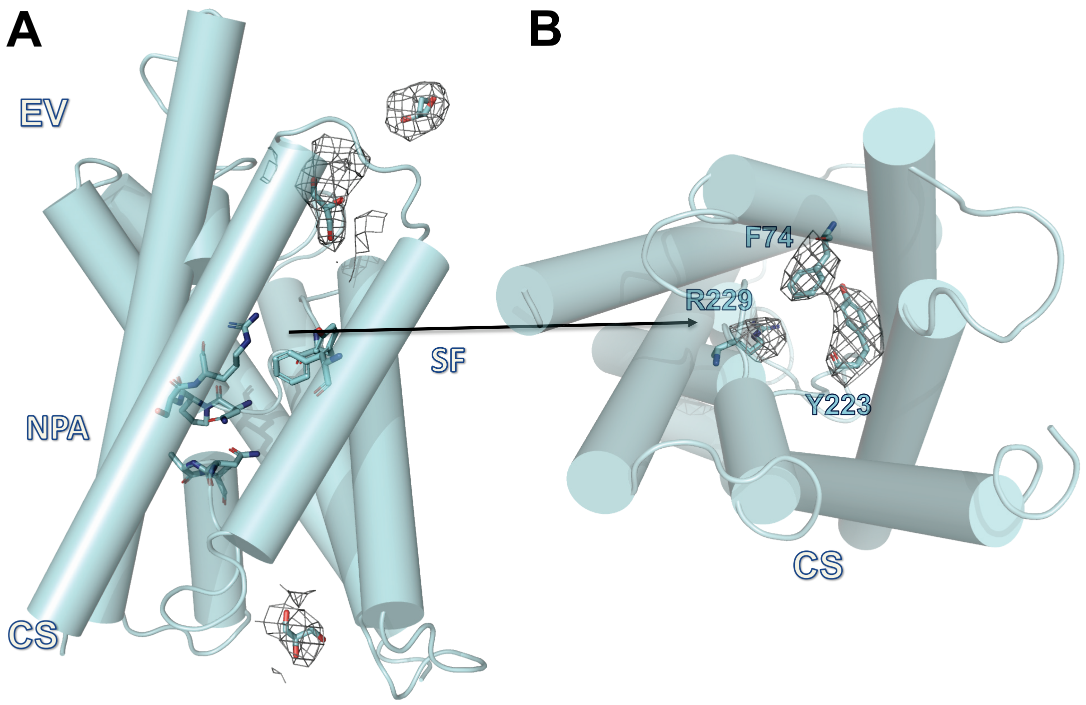

Supplementary Figure S2. 2Fo-Fc omit maps showing selected residues/glycerols of interest. **A**) Side view of a representative AQP7 monomer depicting initial 2Fo-Fc omit map density (contoured at 1 σ**)** for three glycerol molecules (top to bottom: glycerol at the extracellular crystallographic interface, glycerol at the EV, glycerol at the CS). We do not display the glycerol molecule near the NPA restriction site because the final maps are of limited quality, indicating both partial disorder and low occupancy. **B**): The view from the cytosolic side of a representative AQP7 monomeric pore, depicting 2Fo-Fc omit map density (contoured at 3.5 σ**)** for the 'closed state' of the SF region, which comprises residues F74, Y223, and R229.

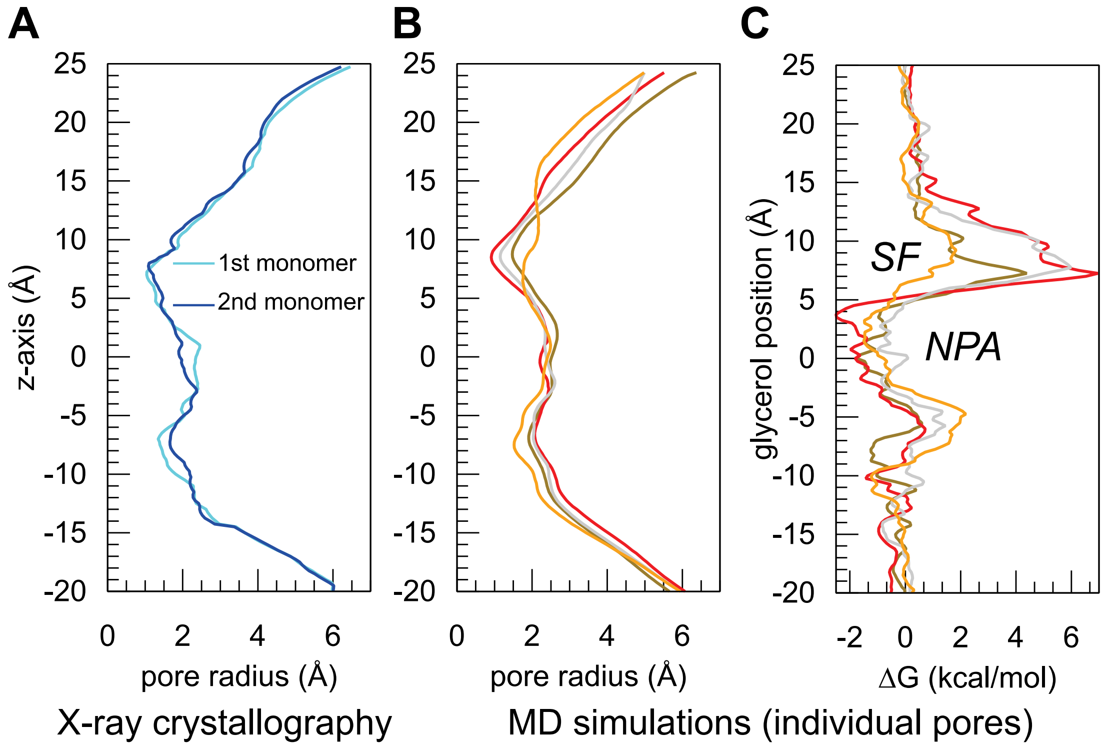

Supplementary Figure S3. A) Pore radius profiles of the individual AQP7 monomers in the X-ray resolved dimer calculated using the HOLE program (Smart et al., 1996). The calculated bottleneck pore radii at the SF region are 1.04 Å for the first monomer (cyan) and 1.08 Å for the second monomer (blue), corresponding the constriction diameters of 2.08 Å and 2.16 Å, respectively. **B**) Pore radius profiles of individual pores of the tetramer averaged over the 200-ns MD equilibration. The average bottleneck pore radii of the SF region range from 0.91 Å (red) to 1.75 Å (orange). **C**) Free energy (ΔG) profiles of glycerol partitioning along the pores (this panel is a reproduction of **Figure 5A** but without the average profile displayed). The pore with the highest maximum ΔG at the SF region corresponds the narrowest constriction diameter of 1.82 Å (red). The one with the lowest maximum ΔG has the widest constriction diameter of 3.5 Å (orange).

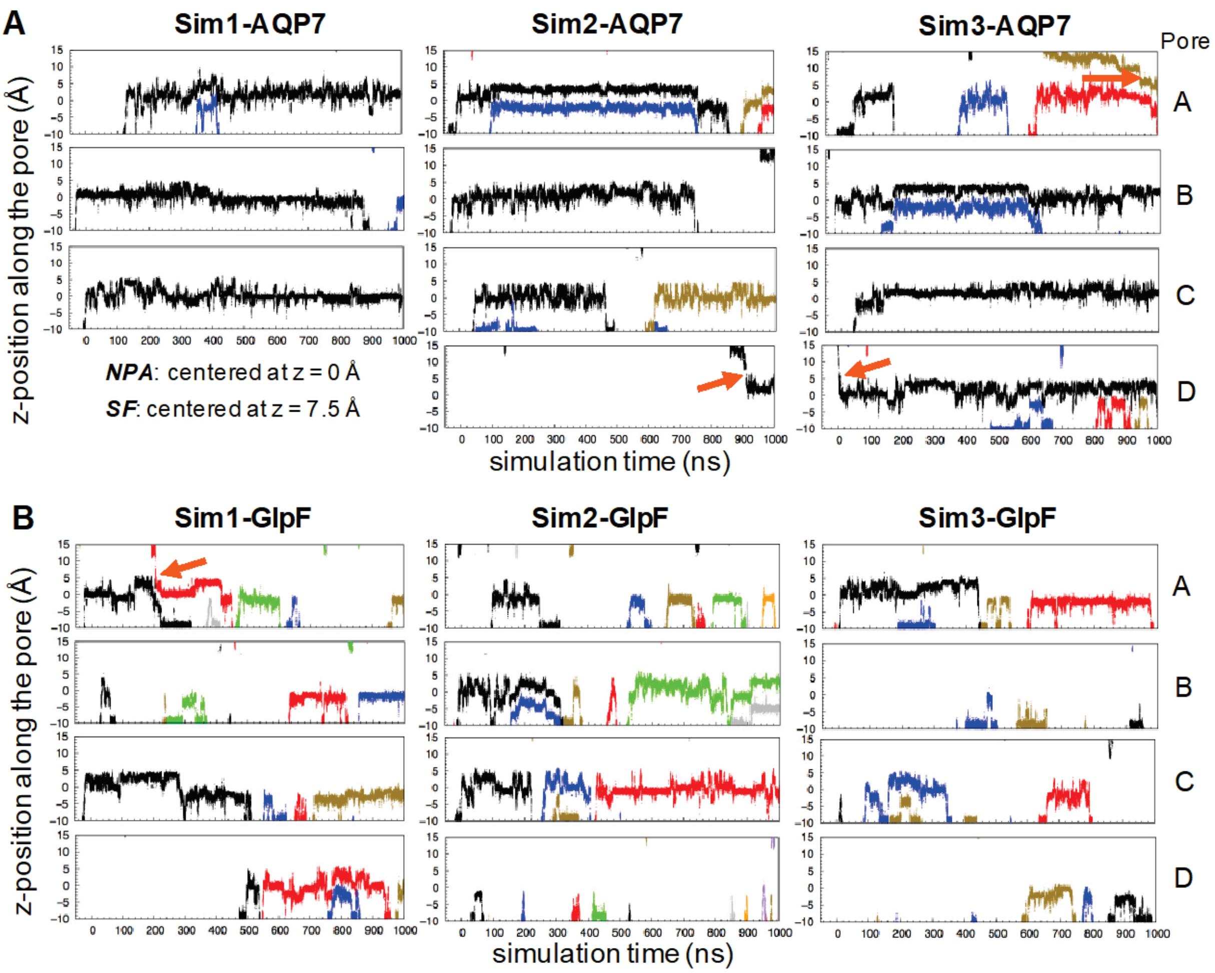

Supplementary Figure S4. Transition events of glycerol molecules between the extracellular side and the NPA constriction site of AQP7 (**A**) and GlpF (**B**) during 1-μs flooding simulations of 200 glycerol molecules. Each line corresponds to an individual glycerol molecule entering the NPA site. Most of the molecules entered the NPA site from the cytoplasmic side (z<0). Of three simulations, only three transition events across the SF from the extracellular side (z>0) took place in AQP7, and only one event took place in GlpF; these events are highlighted by orange arrows.

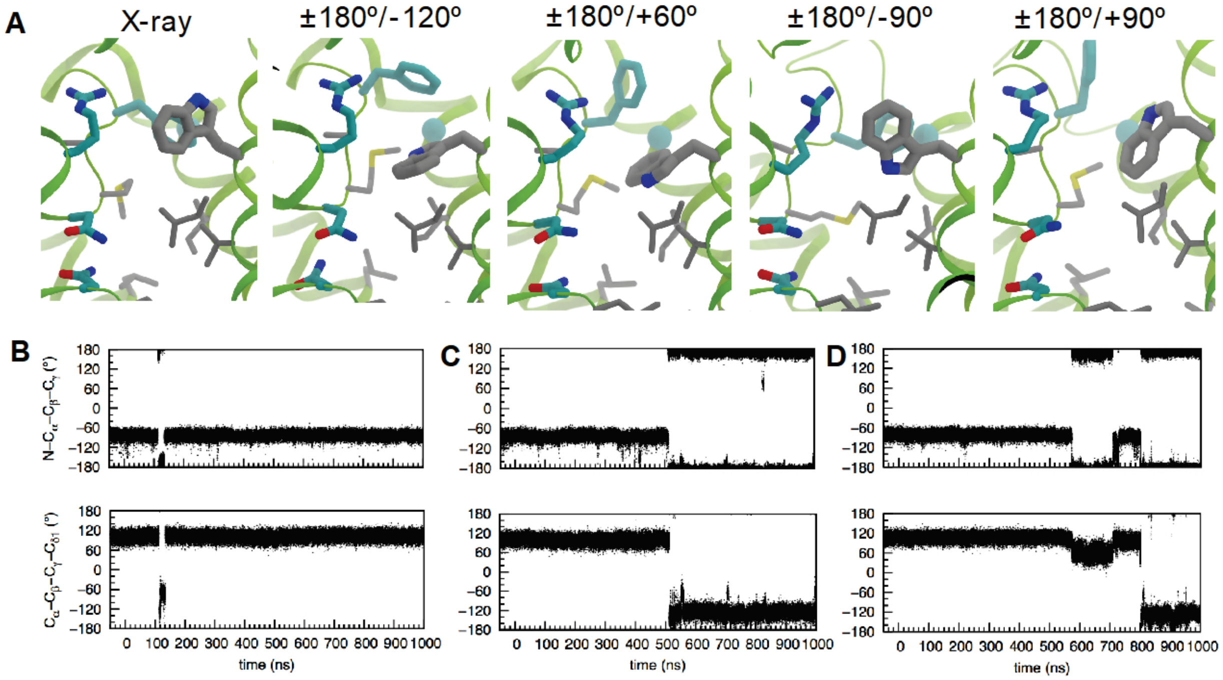

Supplementary Figure S5: Observed side chain conformations of W48 of GlpF (**A**). The X-ray conformation is at $\chi_1 \sim +90^o$ and $\chi_2 \sim +90^o$, where χ_1 and χ_2 correspond to N-C_α-C_β-C_γ and C_α -C_β-C_γ-$C_{\delta 1}$ dihedral angles, respectively. This conformation remained as the major conformation during the simulations. Nevertheless, the simulations were able to capture a -90° rotation of the indole side chain, reflected by the transition of χ_1 from ~ 90° to ±180°. These conformations took placed in Sim1 (**B** & **C**) and Sim3 (d). At $\chi_1 \sim \pm180^o$, when χ_2 were at -120° or +60°, the side chain protruded in the permeation pathway, potentially hindering the passage of water and solutes (**C** & **D**).

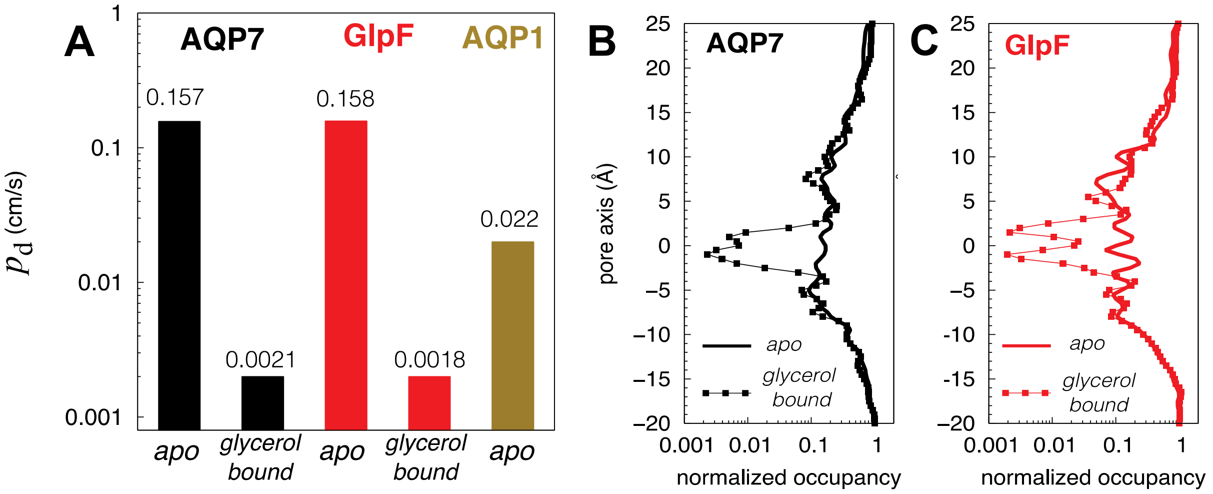

Supplementary Figure S6: Glycerol binding inhibits water diffusion through the monomeric pores. We perform simulations in the apo condition in the absence of glycerol molecules for 200 ns. In the glycerol bound condition, each pore contained one glycerol molecule bound to its NPA region, with the position of the molecule restrained by a harmonic potential located at the most favorable glycerol-binding site. We extended the glycerol bound simulations to 250 ns. Since no glycerol binding in the monomeric pores occurred during a 1-μs flooding simulation of 200 glycerol molecules, we performed only the apo simulation. The diffusive permeability coefficients (P_d), shown in Panel **A** were calculated by normalizing the total number of water permeation events through the monomeric pores, shown in Panel **B** & **C**, over the horizontal area of the protein (~4,000 Å^2) and the concentration of water in the bulk solution (55.5 M). In the absence of glycerol, water permeability in AQP7 and GlpF are ~7-times greater than in AQP1. However, when their monomeric pores were occupied by glycerol molecules, water permeability decreased by ~70 fold and they became ~10-fold less permeable than those of AQP1.

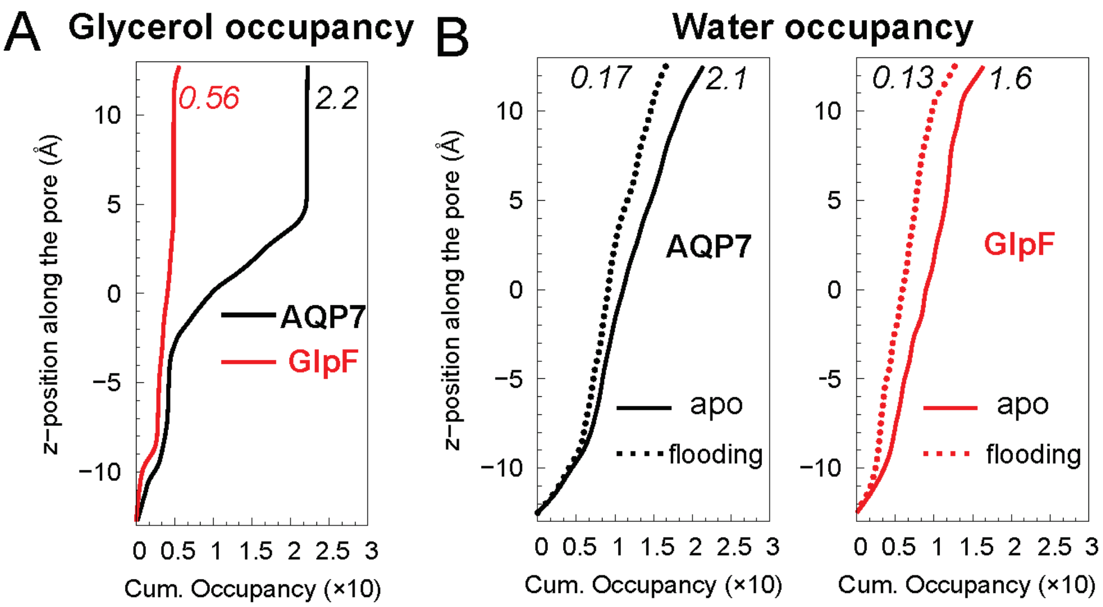

Supplementary Figure S7: Cumulative occupancy of glycerol and H_2O in the AQP7 and GlpF pores. **A**) The cumulative occupancy calculated region spans from z = -12.5 Å to z = +12.5 Å (rugged occupancy and free energy profiles). The probability of glycerol insertion in the AQP7 pore is ~4 fold greater than in GlpF. **B**) For both the 200-ns apo simulations (no glycerol molecules) and the 1050-ns flooding simulations of 200 glycerol molecules, the H_2O occupancy of AQP7 was 1.3 fold greater than in GlpF.

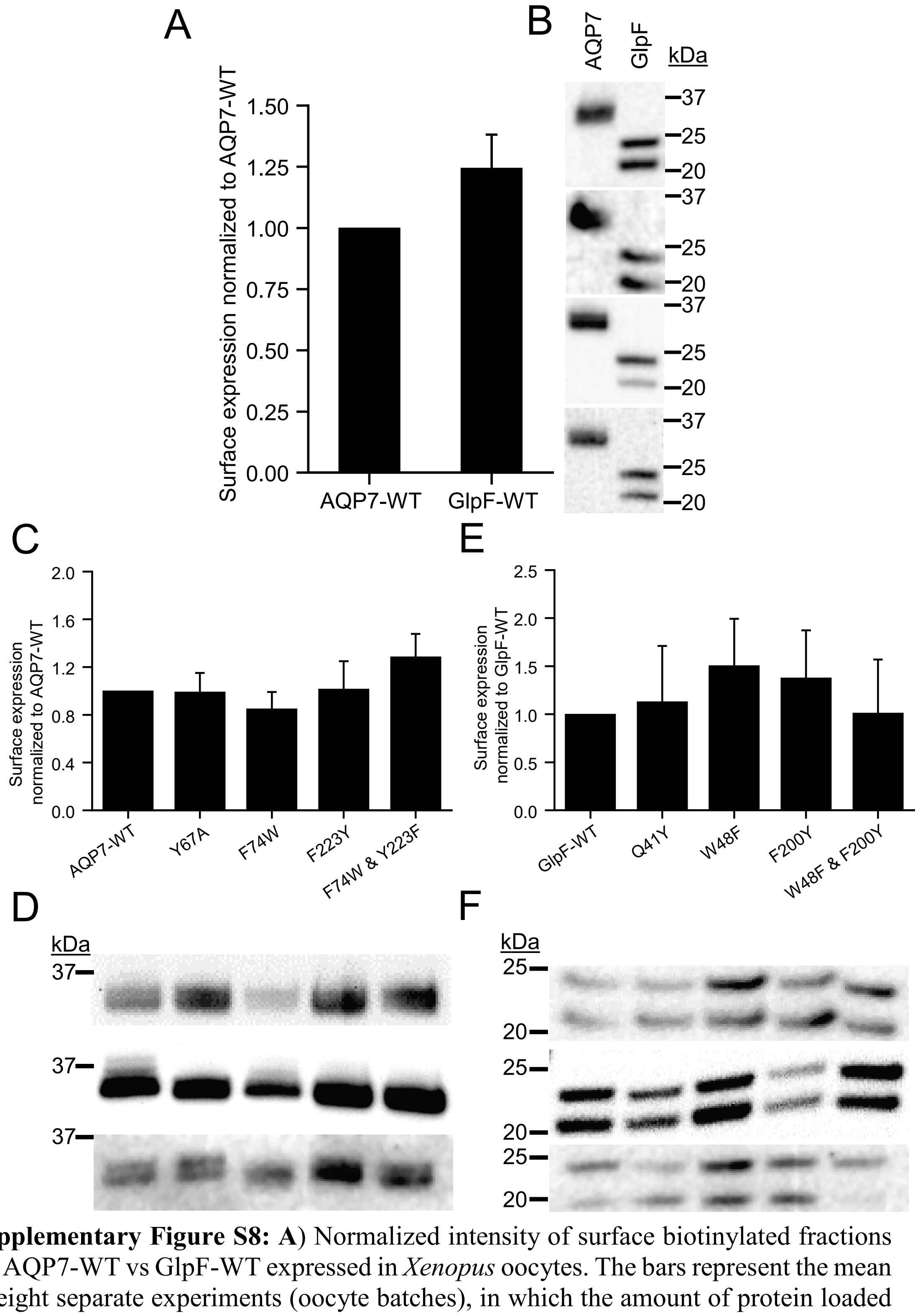

Supplementary Figure S8: A) Normalized intensity of surface biotinylated fractions for AQP7-WT vs GlpF-WT expressed in *Xenopus* oocytes. The bars represent the mean of eight separate experiments (oocyte batches), in which the amount of protein loaded per lane is equivalent to 1 oocyte but comes from a group of 12 oocytes incubated with biotin as described in SI Appendix, Materials and Methods. We normalize the GlpF-WT band intensity to the AQP7-WT band intensity on the same blot from each oocyte batch. **B**) Four representative blots of the AQP7-WT and GlpF-WT surface fraction bands. Surface biotinylation experiments also determine the relative expression at the oocyte plasma membrane of the AQP7 (**C** & **D**) and GlpF (**E** & **F**) mutants compared to their respective WT proteins. Bars represent mean ± S. E. M of six experiments.

Statistics are calculated as described in in SI Appendix, Materials and Methods. Example blots relative surface expression of AQP7-WT and mutant (**D**) and GlpF-WT and mutant (**F**) from three different batches of oocytes are displayed below the bar graphs with the samples loaded in the same lane order as the bar graphs in panels **C** & **E** respectively.

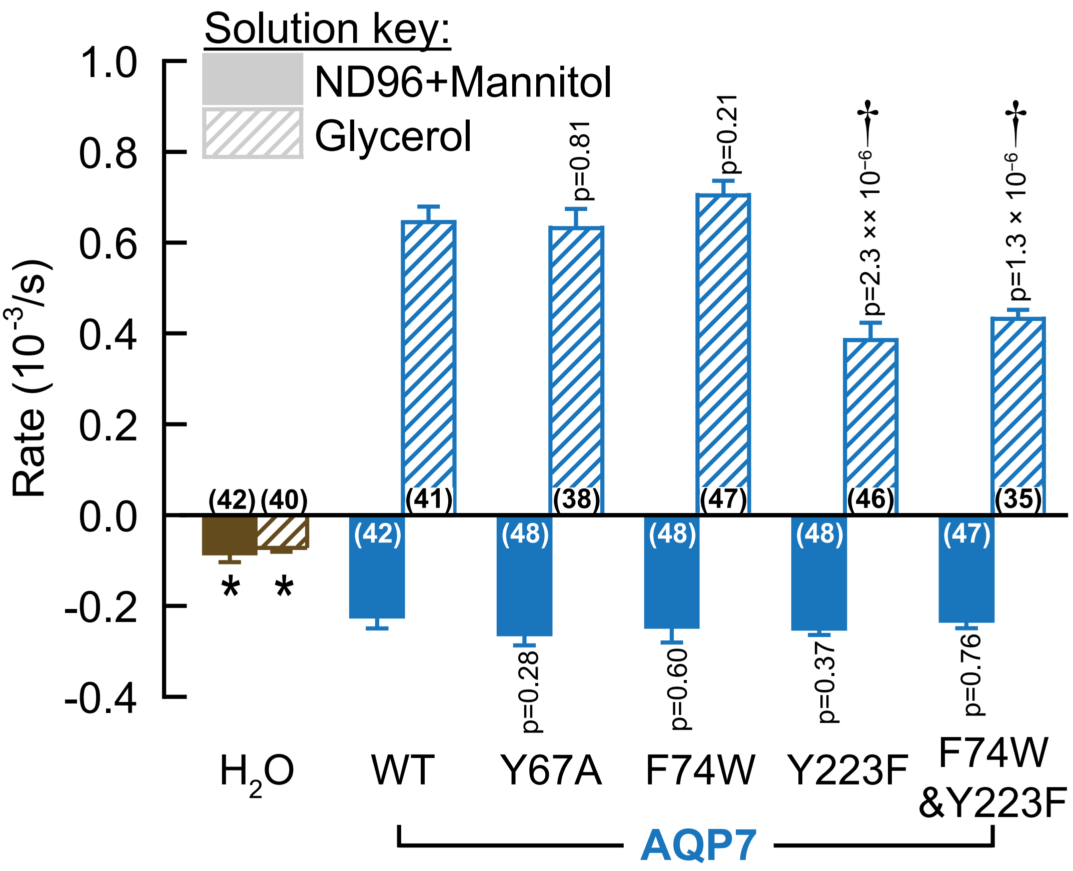

Supplementary Figure S9: Results of an osmotic-shrinking assay for oocytes expressing AQP7 and its variants possessing mutations at the residues indicated when placed in 270 mOsm ND96+Mannitol or glycerol buffers after pre-incubation in normo-osmotic ND96 (195 mOsm) solutions. * denotes that the rate measured for H_2O control oocytes is significantly different than all tested AQP7 variants. † denotes a significant difference compared to WT AQP7. Bars represent mean ± S. E. M. Statistics are calculated as described in Materials and Methods. We harvested oocytes two frogs to perform these experiments.

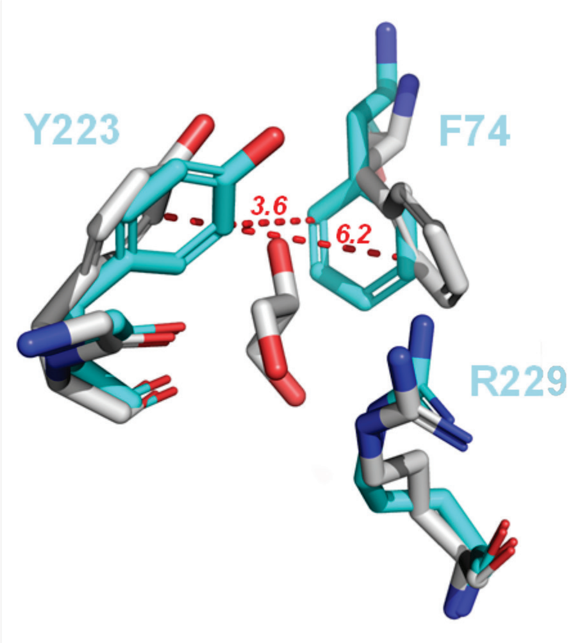

Supplementary Figure S10: Overlay of the selectivity filter (SF) region for AQP7 (‘open state’, grey, from 6QZI, (de Maré et al., 2019)) and AQP7 (‘closed state’, cyan, from our 6N1G structure; see Figure 4) formed by the three residues Y223, F74 and R229. The glycerol present in the ‘open state’ is shown in gray. The structure overlay shows a shift of 2.6 Å for the distance Cε2 – Cδ2 between Y223 and F74.

Supplementary Tables

Supplementary Table S1: Swelling rates, replicates (n), and number of oocyte preparations (Preps) performed for AQP1 oocytes in normo-osmotic buffer plotted in **Figure 2** and **Figure 7**.

[Glycerol]$_o$ (mM)	AQP1			
	Rate (10^{-4}/s)	S. E. M	n	Preps
**0**	0.71	0.28	18	2
**5.625**	0.09	0.13	18	3
**11.25**	0.17	0.16	12	2
**22.5**	0.37	0.19	17	3
**45**	0.19	0.08	10	2
**90**	0.19	0.13	18	3
**130**	0.83	0.56	12	2
**180**	0.77	0.43	12	2

Supplementary Table S2: Swelling rates, replicates (n), and number of oocyte preparations (Preps) performed for AQP7 and AQP7 + AQP1 oocytes in normo-osmotic buffer as plotted in **Figure 2A**.

[Glycerol]$_o$ (mM)	AQP7				AQP7 + AQP1			
	Rate (10^{-4}/s)	S. E. M	n	Preps	Rate (10^{-4}/s)	S. E. M	n	Preps
**0**	0.72	0.28	18	2	0.56	0.22	18	2
**5.625**	0.38	0.25	18	3	0.92	0.28	18	3
**11.25**	0.37	0.14	12	2	1.09	0.26	12	2
**22.5**	0.65	0.15	17	3	1.32	0.22	18	3
**45**	1.30	0.37	12	2	1.99	0.60	12	2
**90**	4.19	0.51	18	3	7.35	1.28	18	3
**130**	5.71	1.00	11	2	13.50	2.37	12	2
**180**	10.10	1.83	10	2	23.50	3.87	12	2

Supplementary Table S3: Swelling rates, replicates (n), and number of oocyte preparations (Preps) performed for GlpF and GlpF + AQP1 oocytes in normo-osmotic buffer as plotted in **Figure 2B**.

[Glycerol]$_o$ (mM)	GlpF				GlpF + AQP1			
	Rate (10^{-4}/s)	S. E. M	n	Preps	Rate (10^{-4}/s)	S. E. M	n	Preps
**0**	0.21	0.09	16	2	0.26	0.10	18	2
**5.625**	0.22	0.09	18	3	0.90	0.35	24	3
**11.25**	0.30	0.11	12	2	0.44	0.07	17	2
**22.5**	0.83	0.33	18	3	0.84	0.17	21	3
**45**	0.51	0.10	12	2	1.73	0.34	12	2
**90**	0.63	0.25	18	3	6.41	2.45	20	3
**130**	1.13	0.32	12	2	6.88	1.18	11	2
**180**	1.27	1.48	12	2	6.90	1.77	12	2

Supplementary Table S4: Swelling rates, replicates (n), and number of oocyte preparations (Preps) performed for AQP7-F74W and AQP7-F74W + AQP1 oocytes in normo-osmotic buffer as plotted in **Figure 7A**.

[Glycerol]$_o$ (mM)	AQP7-F74W				AQP7-F74W + AQP1			
	Rate (10^{-4}/s)	S. E. M	n	Preps	Rate (10^{-4}/s)	S. E. M	n	Preps
**0**	0.08	0.14	18	2	0.81	0.34	18	2
**5.625**	0.19	0.11	18	3	0.25	0.10	18	3
**11.25**	0.78	0.26	12	2	0.86	0.22	12	2
**22.5**	0.96	0.29	18	3	1.42	0.16	17	3
**45**	1.82	0.19	12	2	3.63	0.50	12	2
**90**	2.65	0.52	17	3	9.25	0.71	16	3
**130**	4.92	0.69	12	2	11.00	1.25	11	2
**180**	5.61	1.02	12	2	30.90	3.20	10	2

Supplementary Table S5: Swelling rates, replicates (n), and number of oocyte preparations (Preps) performed for AQP7-Y223F and AQP7-Y223F + AQP1 oocytes in normo-osmotic buffer as plotted in **Figure 7B**.

[Glycerol]$_o$ (mM)	AQP7-Y223F				AQP7-Y223F + AQP1			
	Rate (10^{-4}/s)	S. E. M	n	Preps	Rate (10^{-4}/s)	S. E. M	n	Preps
**0**	0.65	1.06	11	2	0.50	0.36	12	2
**5.625**	-0.22	0.09	12	2	0.21	0.11	12	2
**11.25**	0.26	0.11	6	1	0.54	0.20	6	1
**22.5**	0.33	0.19	12	2	1.01	0.22	12	2
**45**	0.86	0.25	6	1	2.21	0.17	6	1
**90**	2.82	0.24	12	2	3.76	0.72	12	2
**130**	3.69	0.72	12	2	7.91	1.76	12	2
**180**	3.79	0.72	12	2	9.63	1.05	12	2

Supplementary Table S6: Swelling rates, replicates (n), and number of oocyte preparations (Preps) performed for AQP7-F74W & Y223F and AQP7-F74 & Y223F + AQP1 oocytes in normo-osmotic buffer as plotted in **Figure 7C**.

[Glycerol]$_o$ (mM)	AQP7-F74W & Y223F				AQP7-F74W & Y223F + AQP1			
	Rate (10^{-4}/s)	S. E. M	n	Preps	Rate (10^{-4}/s)	S. E. M	n	Preps
**0**	1.08	0.39	12	2	0.49	0.24	8	2
**5.625**	0.20	0.09	12	2	0.30	0.08	12	2
**11.25**	0.46	0.29	12	2	0.54	0.35	10	2
**22.5**	0.36	0.11	6	1	1.13	0.24	12	2
**45**	1.21	0.33	6	1	1.87	0.42	6	1
**90**	3.84	0.33	12	2	8.25	1.00	12	2
**130**	4.80	0.31	12	2	11.10	1.77	11	2
**180**	6.66	0.80	12	2	15.30	2.24	12	2

Supplementary Table S7: Swelling rates, replicates (n), and number of oocyte preparations (Preps) performed for GlpF-W48F and GlpF-W48F + AQP1 oocytes in normo-osmotic buffer as plotted in **Figure 7D**.

[Glycerol]o (mM)	GlpF-W48F				GlpF-W48F + AQP1			
	Rate (10^{-4}/s)	S. E. M	n	Preps	Rate (10^{-4}/s)	S. E. M	n	Preps
**0**	0.50	0.29	11	2	0.11	0.08	17	2
**5.625**	0.64	0.29	12	2	0.43	0.12	24	3
**11.25**	0.41	0.25	6	1	0.09	0.14	18	2
**22.5**	0.10	0.04	12	2	0.10	0.06	26	3
**45**	0.20	0.11	6	1	0.67	0.26	12	2
**90**	0.48	0.33	12	2	0.60	0.11	24	3
**130**	0.05	0.05	12	2	0.71	0.27	18	2
**180**	0.98	1.88	12	2	0.65	0.32	18	2

Supplementary Table S8: Swelling rates, replicates (n), and number of oocyte preparations (Preps) performed for GlpF-F200Y and GlpF- F200Y + AQP1 oocytes in normo-osmotic buffer as plotted in **Figure 7E**.

[Glycerol]o (mM)	GlpF-F200Y				GlpF-F200Y +AQP1			
	Rate (10^{-4}/s)	S. E. M	n	Preps	Rate (10^{-4}/s)	S. E. M	n	Preps
**0**	0.45	0.82	12	2	0.69	0.27	12	2
**5.625**	-0.02	0.20	12	2	0.15	0.16	12	2
**11.25**	0.05	0.05	11	2	0.07	0.06	12	2
**22.5**	0.37	0.16	13	2	0.33	0.10	12	2
**45**	0.71	0.48	6	1	1.24	0.23	6	1
**90**	0.36	0.28	12	2	1.11	0.50	11	2
**130**	0.33	0.15	10	2	0.63	2.15	12	2
**180**	0.63	0.21	12	2	4.19	1.24	11	2

Supplementary Table S9: Swelling rates, replicates (n), and number of oocyte preparations (Preps) performed for GlpF-W48F & F200Y and GlpF-W48F & F200Y + AQP1 oocytes in normo-osmotic buffer as plotted in **Figure 7F**.

[Glycerol]o (mM)	GlpF-W48F & F200Y				GlpF-W48F & F200Y+ AQP1			
	Rate (10^{-4}/s)	S. E. M	n	Preps	Rate (10^{-4}/s)	S. E. M	n	Preps
**0**	0.37	0.13	12	2	0.22	0.13	12	2
**5.625**	0.24	0.10	11	2	0.17	0.13	12	2
**11.25**	0.19	0.09	12	2	0.13	0.14	12	2
**22.5**	0.08	0.09	12	2	0.19	0.11	12	2
**45**	0.32	0.08	6	1	0.31	0.10	6	1
**90**	0.05	0.11	12	2	0.55	0.20	12	2
**130**	0.18	0.15	12	2	0.95	0.40	12	2
**180**	0.66	0.42	12	2	1.42	0.63	12	2

Supplementary Material: Materials and Methods

Molecular biology: Cloning

For protein expression, the human AQP7 (NM_001170) open reading frame is synthesized (Thermo Fisher Scientific) and subcloned into the pIEX-Bac-3 plasmid (MilliporeSigma) using standard molecular biology techniques. For *Xenopus* oocyte expression, the open reading frames for myc-HIS tagged human AQP7 and GlpF (translated sequence identical to WP_000084268 but codon optimized for human) are synthesized (Genscript) and sub-cloned into the pGH19 vector flanked by the 5′- and 3′- UTR of the *Xenopus* β-globin gene (Toye et al., 2006).

X-ray crystallography

Protein purification and expression

Suspension Sf9 cells are cultured in ESF 921 Insect Cell Culture Medium (Expression Systems, CA). Healthy cells are split at density of $3.5x10^6$ cell/ml and infected with AQP7 baculovirus and then shaken at 27°C and 130 rpm for 2 days before harvesting by centrifugation. The cells are resuspended in breaking buffer (5 mM Tris pH 8.0, 100 mM NaCl, 1mM PMSF, 1 mM EDTA), supplemented with protease inhibitor tablets (Sigma-Aldrich). Cells are broken with a 10 ml Dounce homogenizer (20 passes with loose and 20 passes with tight pestle) and immediately centrifuged at 113,000 × *g* to collect the membrane fraction. The resulting pellet is resuspended in homogenization buffer (4 M urea, 5 mL Tris-HCl [pH 8.0], 5 mM EDTA, 5 mM EGTA, 0.01% NaN_3). After resuspension, the pellet is homogenized (20 passes), then centrifuged at 125,000 × *g*, 60 min, 4°C to collect the membrane fraction. The resulting pellet is resuspended, homogenized and centrifuged twice more with 50 mL of 20 mM NaOH, and subsequently with pH restore buffer (5 mM Tris [pH 8.0], 100 mM NaCl, 0.03% NaN_3). The resulting membrane fraction is resuspended in buffer B (20 mL $NaPO_4$ [pH 7.8], 200 mM NaCl, 10% glycerol, 0.01% NaN_3) if stored. For

solubilization, the membranes are first resuspended in solubilization buffer (20 mM $NaPO_4$ [pH 7.8], 200 mM NaCl, 2 mM β-mercaptoethanol, 10% (v/v) glycerol, 0.03% (w/v) NaN_3, 2.5% (w/v) octyl glucoside [Anatrace], 0.2% (w/v) decyl maltoside (DM) [Anatrace]). Next, the solution is homogenized (10 passes), then incubated at room temperature for 30 min. The unsolubilized material is removed by centrifugation at 150,000 × g, 60 min, 4°C. Purification is done by immobilized metal affinity chromatography (IMAC) using Ni-NTA resin (Qiagen). We wash the His-tagged protein constructs with wash buffer (20 mM Tris [pH 7.8], 200 mM NaCl, 5% glycerol, 2 mM β-mercaptoethnol, 55 mM imidazole, 0.3% DM), prior to elution with elution buffer (20 mM Tris [pH 8.0], 250 mM NaCl, 0.01% NaN_3, 3 mM β-mercaptoethnol, 350 mM imidazole, 0.4% DM). We assess sample purity by SDS-PAGE. We concentrate the eluted protein to a volume of 450 µl and further purify by size exclusion chromatography (Superdex S200, Amersham) in 20 mM Tris-HCl pH 7.8 containing 200 mM NaCl, 2 mM β-mercaptoethnol and 0.3% DM. The chromatograms reveal 2 separate peaks corresponding to the octameric and tetrameric assemblies of AQP7. We collect both peaks separately and concentrate to 10-12 mg/ml. Immediately prior to crystallization trials, the concentrated fractions are centrifuged at 150,000-× g for 60 min in order to remove any aggregated micro particles,

Crystallization and data collection

We grow crystals at 18°C using the sitting drop vapor diffusion method. The reservoir solution contained 30% w/v polyethylene glycol 400, 100 mM MOPS pH 7.0, 100 mM NaCl. We prepare drops were by mixing 0.8 µl reservoir solution and 0.8 µl protein solution containing 14 mg/ml protein, 0.2 M NaCl, 0.4% DM and 20 mM Tris-HCl, pH 7.5. Cubic shaped crystals form within 3-4 days and are flash-cooled directly in liquid nitrogen for storage. We collect X-ray data at the Advanced Photon Source

beamline NE-CAT ID-24-C of Cornell University at Argonne National Laboratory, and process using HKL2000 (Otwinowski and Minor, 1997) and Scalepack (**Table 1**). The best crystal diffracts to the 3.95 Å resolution shell with an overall R_{pim} of 2.9%. We determine the space group as $F4_132$ with a solvent content of 64%, resulting in an occupancy of four monomers per asymmetric unit. The unit-cell parameters are a = b = c = 385.66 Å. The data set is 96.7% complete to 3.95 Å resolution.

Structure determination

We obtain initial phases for the core structure of AQP7 by molecular replacement using Phaser (McCoy et al., 2005). We assemble a search model with Swissmodel using the existing homologous structural model of aquaporin-5 (PDB-code: 3D9S) (Horsefield et al., 2008). The N- and C-terminal region as well as loop regions of this model are truncated as they were thought to be conformationally flexible. Based on the initial solution found (Z-score = 12.4, R_{free} = 40.2%), we use the model for further manual building and refinement using programs Coot (Emsley et al., 2010) and refmac5 (Murshudov et al., 2011, 5). After several refinement cycles and model building, we place the main chains in the missing loop and N-terminal regions. We show data collection and refinement statistics in **Table 1**. We calculate the difference in the GlpF NPA-NPA motif vs the AQP7 NPS-NAA motif buried surface area by subtracting the combined surface area of the individual NPA and NPA or NPS and NAA regions (from GlpF and AQP7 respectively) from the sum of the buried surface area of the individual regions. All values were calculated using the CCP4 program AREAIMOL (Winn et al., 2011). Structure superposition was performed utilizing the CCP4 program, Superpose, which performs structural alignment based on secondary structure matching. An initial alignment based upon matching of secondary structure graphs is then improved with an iterative 3-D alignment of protein backbone C-alpha atoms between the two structures.

This approach gives good results in global alignment when proteins have similar structures but is not biased by divergence in sequence (Krissinel and Henrick, 2004).

Molecular Dynamics Simulations

Setup and general simulation protocols

The human AQP7 is the primary protein simulated in the present study, so we use the 3.9 Å resolution crystal structure reported here as the structural model. The tetrameric protein is generated using the provided transformation matrix. The program Dowser (Zhang and Hermans, 1996) is used to model internal water, yielding 75 water molecules. To provide structural support for the function of AQP7 in terms of glycerol permeability, *E. coli* GlpF (glycerol facilitator protein) and bovine AQP1, are also studied. We use the 2.2Å crystal structure of GlpF (PDB entry 1FX8) (Fu et al., 2000) with glycerol molecules bound to the NPA region, selectivity filter (SF) and extracellular vestibule as the structural model. This GlpF structure contains 444 crystal water molecules. For AQP1, which is glycerol-impermeable, the 2.2Å crystal structure (PDB entry 1J4N) (Sui et al., 2001) including 456 crystal water molecules is used as the initial model.

To construct the membrane-bound models, we use the OPM database (Lomize et al., 2006, 2012) to predict the orientation of each protein in the membrane and the CHARMM-GUI webserver (Jo et al., 2008) to insert each protein in a membrane patch. POPC lipids are used for AQP7 and AQP1, while POPE lipids are used for GlpF. We use SOLVATE and AUTOIONIZE plugins of VMD (Humphrey et al., 1996) to solvate the membrane-embedded models with TIP3P water and ionize with 0.2 M NaCl. The AQP7 system comprises 118,700 atoms (78 Na^+ and 90 Cl^- ions, 351 lipids and 21,000 water molecules). The GlpF system comprises ~122,900 atoms (80 Na^+ and 84 Cl^- ions, 351 lipids and 21,100 water molecules). The AQP1 system comprises ~105,000 atoms (72

Na^+ and 84 Cl^- ions, 247 lipids and 19,400 water molecules).

All simulations are performed using NAMD (Phillips et al., 2005) with a time step of 2 fs and the CHARMM36 force field (MacKerell et al., 1998; Klauda et al., 2010; Best et al., 2012). We use periodic boundary conditions (PBC) throughout the simulations. All bonds involving hydrogen atoms are kept rigid using the SHAKE algorithm (Ryckaert et al., 1977). To evaluate long-rang electrostatic interactions, we use the particle mesh Ewald method (Darden et al., 1993) with a grid density of 1 $Å^{-3}$. We set the 12 Å distance as the cutoff for van der Waals interactions. Langevin dynamics maintains the temperature at 310K by with a damping coefficient of 1 ps^{-1}. We use the Nosé-Hoover Langevin piston method (Martyna et al., 1994; Feller et al., 1995) with a piston period of 200 fs to maintain the pressure at 1 atm.

Each set of MD simulations comprises the following pre-equilibration steps: (1) 0.5 ns with harmonic restraints of k = 1 kcal/mol/$Å^2$ applied to non-hydrogen atoms of the proteins, internal water and X-ray glycerol molecules; (2) 0.5 ns with only the backbone atoms of the protein and the non-hydrogen atoms of the internal water and glycerol molecules spatially restrained; (3) 2 ns with the C_α atoms of the protein and the non-hydrogen atoms of the internal water and glycerol molecules spatially restrained. We perform energy minimization for 1,000 steps in the beginning of each step. Then, we perform at least 20 ns of unrestrained relaxation MD for each of the membrane-embedded protein complexes before the production simulations, which include umbrella sampling (for AQP7 and GlpF) and flooding simulations (for AQP7, GlpF and AQP1). Note that the pre-equilibrations of AQP1 contain no glycerol molecules.

Umbrella sampling simulations

We employ the umbrella sampling (US) technique (Torrie and Valleau, 1977; Roux,

1995) to calculate the free energy (ΔG) profiles describing the translocation of a glycerol molecule through each of the four monomeric pores of AQP7 and GlpF. We simulate one glycerol molecule along a defined section of each pore, so the calculations contain a total of 4 glycerol molecules for AQP7. For GlpF, the glycerol molecules dissociate from pores C and D completely during the 20-ns relaxation phase, so we simulate the glycerol molecules only along pores A and B. The starting configurations for the US simulations are generated using constant-velocity steered molecular dynamics (SMD) simulations (Izrailev et al., 1998). We use the center of mass of alpha carbons of amino acids within the NPA region (N94, N226, M47, V78, L182 V210 for AQP7, and N68, N203, L21, V52, L159 and I187 for GlpF) as the reference position set to z = 0. During SMD, the glycerol molecule is pulled from the reference either toward the extracellular surface or toward the cytoplasmic surface, to span from z = -20 Å to z = +20 Å. We start the SMD simulations using the last snapshot of the 20 ns unrestrained relaxation run. The pulling velocity is 2 Å/ns. The harmonic potential applied to restrain the glycerol molecule (heavy atoms) to the reference is at k = 20 kcal/mol/Å^2.

The US simulation comprises 91 0.5-Å windows. We pre-equilibrate each window with the applied k of 20 kcal/mol/Å^2 for 0.5 ns. The starting structures are extracted from the SMD runs and simulated for 10 ns with k = 5 or 10 kcal/mol/Å^2 depending on the position of the molecule. Thus, the effective US simulation time is 960 ns for each system. We construct the ΔG profiles using the weighted histogram analysis method (WHAM code was implemented by Professor Alan Grossfield at the University of Rochester Medical Center http://membrane:urmc:rochester:edu/content/wham)(Kumar et al., 1992) with 0.25-Å histogram bins analyzed from the last 5-ns trajectory of each US simulation the results of which appeared converged. The profiles are normalized

with respect to the hydration ΔG of glycerol in the aqueous solution.

Flooding simulations

In order to increase the likelihood of capturing glycerol binding to the channels during unbiased simulations, three independent simulations are performed for each GlpF and AQP7 in their membrane-embedded form. In the beginning of each simulation we add 200 glycerol molecules to the simulation box containing the membrane-embedded tetrameric complex of either GlpF or AQP7. We initially distribute the glycerol molecules in the aqueous solution. Since we perform the simulations under a periodic boundary condition, the concentrations of glycerol are the same on both sides of the membrane. We use the final structure of the 20-ns relaxation run as the initial structure. We remove all crystallized glycerol molecules. With respect to the density of water, these 200 glycerol molecules constitute a concentration of 625 mM. Each simulation lasts 1,050 ns, so the collective simulation time for the two proteins is 6,300 ns. The last 1,000-ns portion of the trajectories from the three simulations are collectively used for the calculation of glycerol occupancy using the VolMap plugin of VMD (Humphrey et al., 1996). We also perform one flooding simulation on AQP1, which is not permeable to glycerol, as a negative control, and the simulation time was 1,050 ns.

We calculate the cumulative occupancy for glycerol and H_2O in the region that spans from z = -12.5 Å to z = +12.5 Å (rugged occupancy and free energy profiles). We calculate the cumulative occupancy K from the free energy profiles in **Figure 5** as follows.

$$K = \int_{-12.5}^{+12.5} e^{-\Delta G(z)/RT}\, dz,$$

K is normalized by the number of bins (51 total) covering the region. K is related to

the diffusive permeability coefficient P_d, which is

$$P_d = \frac{KD}{h}$$

where D corresponds to the overall diffusion coefficient and h is the path length.

Functional Assays

cRNA synthesis for *Xenopus* oocyte expression

We synthesize capped mRNA *in vitro* with the T7 message Machine kit (Ambion, Austin, TX) from cDNA linearized at the Not I restriction site. We purify the cRNA using the RNeasy MinElute Cleanup Kit (QIAgen, Valencia, CA, USA), and determine the concentration by ultraviolet absorbance. We determine the functional activity of myc-HIS tagged AQP7 to not be significantly different from non-tagged AQP7 or a previously described AQP7 construct transcribed from a different *Xenopus* expression plasmid (Geyer et al., 2013).

Solutions

To make the OR3 oocyte maintenance medium, we add 1 sachet of powdered Leibovitz's L-15 medium with glutamine (Cat. No. 41300-039; Thermo Scientific) to 1.5 L double distilled H_2O. Add 200 U penicillin, 2,000 μg streptomycin, and 1.785 g HEPES. We adjust pH to 7.50 with NaOH and adjust volume with H_2O until the osmolarity is 195 ± 5 mOsm.

The ND96 solution contains the following (in mM): 93.5 NaCl, 2 KCl, 1 $MgCl_2$, 1.8 $CaCl_2$, and 5 HEPES (titrated to pH 7.50 with NaOH and osmolality of 195 mOsm) (Musa-Aziz et al., 2010). In the case of P_f assays, we swell oocytes in ND51, a hypotonic variant of ND96 set to 105 mOsm by omitting 45 mM NaCl from the solution (pH 7.50). 105 mOsm NaCl or glycerol solutions also contain 5 mM HEPES (pH 7.50). For isosmotic swelling assays, 2.81, 5.63, 11.25, 22.5 45, 65, or 90 mM NaCl in ND96 is substituted for 5.62, 11.25, 22.5, 45, 90, 130, or 180 mM glycerol respectively. We

confirm osmolality ± 2 mOsm with an osmometer (Wescor model 5520). For oocyte shrinking assays, we increase the osmolality of ND96 to 270 mOsm through the addition of mannitol; the 270 mOsm NaCl or glycerol solutions also contain 5 mM HEPES (pH set at 7.50 with 2.5 mM NaOH). For [^{3}H]-glycerol uptake assays, we substitute 90 mM total glycerol for 45 mM NaCl in the ND96 solution and add 5 μCi [^{3}H]-glycerol (0.8 Ci/mmol) to 200 μl of this solution.

Ethical approval and animal procedures

The protocols for housing and handling of *Xenopus* laevis (NASCO Inc., Fort Atkinson, WI, USA), are approved by the Institutional Animal Care and Use Committee at Case Western Reserve University. In brief, we anesthetize each *Xenopus* by immersion in a solution of 0.2% Tricaine (Sigma-Aldrich). We remove the animal from the solution when it becomes unresponsive to touch, and surgically extract the ovaries. We euthanize the animal prior to recovery from anesthesia by cardiac excision.

Injection of *Xenopus* oocytes with cRNA

Stage V-VI oocytes from *Xenopus* laevis are isolated as described previously (Parker et al., 2012) and then stored until use at 18°C in OR3 medium (Musa-Aziz et al., 2010). The next day, oocytes are injected with 25 nl of cRNA (0.5 ng/nl) or an equal volume of deionized water (Ambion) as a control. Sterile pipettes used for injecting water or cRNA, have tip diameters of 20–30 μm, and are backfilled with mineral oil (Sigma-Aldrich) and connected to a Nanoject II positive-displacement injector (Drummond Scientific, Broomall, PA) or a Roboinject automated robot (Multi Channel Systems). Following injection, the oocytes are stored, at 18°C in OR3 medium, and used in experiments 3–4 days after injection.

Oocyte swelling and shrinking assays.

To determine the osmotic water permeability (P_f), we use a volumetric swelling

assay (Preston et al., 1993; Chandy et al., 1997; Virkki et al., 2002). Before all assays commence, oocytes are transferred from OR3 maintenance medium into ND96 (195 mOsm) and allowed 30 min to equilibrate. We then transfer the oocytes in groups of 6 into a Petri dish that contains a hypotonic ND96 solution (see "solutions") to create a +90 mOsm gradient between the inside and the outside of the oocyte. We immediately begin to acquire digital images at 1 s intervals for a total time of 60 s. From the time course of the projection area of the oocyte (Musa-Aziz et al., 2009), we compute P_f (Preston et al., 1993). To account for the invaginations of the oocyte, we assumed the oocyte to be a sphere with a surface area (S) eightfold greater than that of the idealized sphere (Chandy et al., 1997). By subtracting mean P_f values of day-matched H_2O injected control oocytes from the P_f values of individual AQP-expressing oocytes, we obtain channel-dependent P_f values (P_f*), which reflect "functional expression"–the product of surface-membrane abundance and intrinsic (or per-molecule) activity (Geyer et al., 2013, 0–9).

To determine the rate of swelling due to glycerol, or water + glycerol, in isosmotic swelling assays, it is necessary to provide an unhindered pathway for water influx (Hansen et al., 2002; Beitz et al., 2004). For this purpose, we co-inject oocytes in these assays with 25 nl of human AQP1 (NM_198098) cRNA (0.5 ng/nl). Control oocytes not co-expressing AQP1 are co-injected with 25 nl H_2O. The assay commences when we transfer the oocytes in groups of 6 into a Petri dish that contains an isosmotic ND96+Glycerol solution (see "solutions") and acquire digital images at 1 s intervals for a total time of 60 s. The time course of the projection area of the oocyte is then calculated.

For oocyte shrinking assays, we place the oocytes in 270 mOsm solutions (see "solutions") and measure the time course of the projection area as described above.

Measurement of glycerol permeability of oocytes.

[^{3}H]-glycerol uptake assays. Uptake of [^{3}H]-glycerol (0.8 Ci/mmol) is carried out on groups of 10 oocytes incubated in 200 µl of ND96 buffer containing 5 µCi of the radiolabeled glycerol (nonradioactive glycerol was added to give a 90 mM final concentration) at room temperature. After 10 min, we wash the oocytes rapidly three times in ice-cold ND96 buffer, and then dissolve individual oocytes in 5% SDS for scintillation counting.

Surface biotinylation of plasma-membrane proteins

Biotinylation is performed as previously described (Lee et al., 2012), using the Cell Surface Protein Isolation Kit (Pierce), according to the manufacturer's instructions. We incubate groups of 12 oocytes (in triplicate for each cRNA type injected) for 1 h at 4°C in PBS (diluted to 200 mOsmol/kg H_2O) that contain 0.24 mg/ml of the biotinylation reagent Sulfo-NHS-SS-biotin. Subsequently, we quench the nonreacted biotinylation reagent and disrupted cells by trituration in 120 µl "lysis buffer" that contains Tris-buffered saline, 1% Triton X-100 and protease inhibitors (Roche Applied Biosciences, Indianapolis, IN). The insoluble fraction is pelleted by centrifugation (735 × g for 10 min at 4°C) and the supernatant is then passed through a 0.45 µM Spin-X centrifuge tube filter (Thomas Scientific, Swedesboro, NJ) to clear the oocyte yolk. A 25 µl aliquot of "total oocyte protein" is set aside for analysis by western blot. We incubate the remaining homogenate in the kit-provided mini-column for 1 h with neutravidin agarose. We clear non-bound protein (i.e., non-biotinylated protein) from the column with 3 × 500 µl washes with lysis buffer. Finally, we elute bound, biotinylated protein ("surface fraction") from the column with 95 µl SDS sample buffer that contains 50 mM DTT. Protein is resolved by SDS-PAGE on Novex 10% Bis-Tris gels (Invitrogen), transferred onto polyvinylidene difluoride membranes using the iBlot dry blotting system (Invitrogen), and immunoblotted using anti-human c-myc antibody (Clone

9E10; MAB3696, R&D systems, Minneapolis) antibody diluted 1:2500, followed by a horseradish peroxidase-conjugated goat-anti-mouse polyclonal antibody diluted 1:2500 (Sigma-Aldrich). We use ECL Plus reagents (Pierce) to develop western blots and the signals are detected and imaged using a Fluorchem E imager (Protein Simple, Santa Clara, CA).

References

Beitz, E., Pavlovic-Djuranovic, S., Yasui, M., Agre, P., and Schultz, J. E. (2004). Molecular dissection of water and glycerol permeability of the aquaglyceroporin from Plasmodium falciparum by mutational analysis. *Proc Natl Acad Sci U S A* 101, 1153–1158. doi:10.1073/pnas.0307295101.

Best, R. B., Zhu, X., Shim, J., Lopes, P. E. M., Mittal, J., Feig, M., et al. (2012). Optimization of the additive CHARMM all-atom protein force field targeting improved sampling of the backbone φ, ψ and side-chain χ(1) and χ(2) dihedral angles. *J Chem Theory Comput* 8, 3257–3273. doi:10.1021/ct300400x.

Chandy, G., Zampighi, G. A., Kreman, M., and Hall, J. E. (1997). Comparison of the water transporting properties of MIP and AQP1. *J. Membr. Biol.* 159, 29–39.

Darden, T., York, D., and Pedersen, L. (1993). Particle mesh Ewald: An N·log(N) method for Ewald sums in large systems. *The Journal of Chemical Physics* 98, 10089–10092. doi:doi:10.1063/1.464397.

de Maré, S. W., Venskutonytė, R., Eltschkner, S., de Groot, B. L., and Lindkvist-Petersson, K. (2019). Structural Basis for Glycerol Efflux and Selectivity of Human Aquaporin 7. *Structure*. doi:10.1016/j.str.2019.11.011.

Emsley, P., Lohkamp, B., Scott, W. G., and Cowtan, K. (2010). Features and development of Coot. *Acta Crystallogr. D Biol. Crystallogr.* 66, 486–501. doi:10.1107/S0907444910007493.

Feller, S. E., Zhang, Y., Pastor, R. W., and Brooks, B. R. (1995). Constant pressure molecular dynamics simulation: The Langevin piston method. *The Journal of Chemical Physics* 103, 4613–4621. doi:doi:10.1063/1.470648.

Fu, D., Libson, A., Miercke, L. J., Weitzman, C., Nollert, P., Krucinski, J., et al. (2000). Structure of a glycerol-conducting channel and the basis for its selectivity. *Science* 290, 481–486.

Geyer, R. R., Musa-Aziz, R., Qin, X., and Boron, W. F. (2013). Relative CO_2/NH_3 selectivities of mammalian aquaporins 0-9. *Am. J. Physiol., Cell Physiol.* 304, C985-994. doi:10.1152/ajpcell.00033.2013.

Hansen, M., Kun, J. F. J., Schultz, J. E., and Beitz, E. (2002). A single, bi-functional aquaglyceroporin in blood-stage Plasmodium falciparum malaria parasites. *J. Biol. Chem.* 277, 4874–4882. doi:10.1074/jbc.M110683200.

Horsefield, R., Nordén, K., Fellert, M., Backmark, A., Törnroth-Horsefield, S., Terwisscha van Scheltinga, A. C., et al. (2008). High-resolution x-ray structure of human aquaporin 5. *Proc. Natl. Acad. Sci. U.S.A.* 105, 13327–13332. doi:10.1073/pnas.0801466105.

Humphrey, W., Dalke, A., and Schulten, K. (1996). VMD: Visual molecular dynamics. *Journal of Molecular Graphics* 14, 33–38. doi:10.1016/0263-7855(96)00018-5.

Izrailev, S., Stepaniants, S., Isralewitz, B., Isralewitz, B., Kosztin, D., Lu, H., et al. (1998). "Steered Molecular Dynamics," in *Computational Molecular Dynamics: Challenges, Methods, Ideas* Lecture Notes in Computational Science and Engineering. (Berlin: Springer-Verlag), 39–65. Available at: http://www.springer.com/us/book/9783540632429 [Accessed June 19, 2017].

Jo, S., Kim, T., Iyer, V. G., and Im, W. (2008). CHARMM-GUI: a web-based graphical user interface for CHARMM. *J Comput Chem* 29, 1859–1865. doi:10.1002/jcc.20945.

Klauda, J. B., Venable, R. M., Freites, J. A., O'Connor, J. W., Tobias, D. J., Mondragon-Ramirez, C., et al. (2010). Update of the CHARMM all-atom additive force field for lipids: validation on six lipid types. *J Phys Chem B* 114, 7830–7843. doi:10.1021/jp101759q.

Krissinel, E., and Henrick, K. (2004). Secondary-structure matching (SSM), a new tool for fast protein structure alignment in three dimensions. *Acta Crystallogr. D Biol. Crystallogr.* 60, 2256–2268. doi:10.1107/S0907444904026460.

Kumar, S., Rosenberg, J. M., Bouzida, D., Swendsen, R. H., and Kollman, P. A. (1992). THE weighted histogram analysis method for free-energy calculations on biomolecules. I. The method. *Journal of Computational Chemistry* 13, 1011–1021. doi:10.1002/jcc.540130812.

Lee, S.-K., Boron, W. F., and Parker, M. D. (2012). Relief of autoinhibition of the electrogenic Na-HCO_3 cotransporter NBCe1-B: role of IRBIT vs.amino-terminal truncation. *Am. J. Physiol., Cell Physiol.* 302, C518-526. doi:10.1152/ajpcell.00352.2011.

Lomize, M. A., Lomize, A. L., Pogozheva, I. D., and Mosberg, H. I. (2006). OPM: orientations of proteins in membranes database. *Bioinformatics* 22, 623–625. doi:10.1093/bioinformatics/btk023.

Lomize, M. A., Pogozheva, I. D., Joo, H., Mosberg, H. I., and Lomize, A. L. (2012). OPM database and PPM web server: resources for positioning of proteins in membranes. *Nucleic Acids Res.* 40, D370-376. doi:10.1093/nar/gkr703.

MacKerell, Bashford, D., Bellott, Dunbrack, Evanseck, J. D., Field, M. J., et al. (1998). All-Atom Empirical Potential for Molecular Modeling and Dynamics Studies of Proteins†. *J. Phys. Chem. B* 102, 3586–3616. doi:10.1021/jp973084f.

Martyna, G. J., Tobias, D. J., and Klein, M. L. (1994). Constant pressure molecular dynamics algorithms. *The Journal of Chemical Physics* 101, 4177–4189. doi:10.1063/1.467468.

McCoy, A. J., Grosse-Kunstleve, R. W., Storoni, L. C., and Read, R. J. (2005). Likelihood-enhanced fast translation functions. *Acta Crystallogr. D Biol. Crystallogr.* 61, 458–464. doi:10.1107/S0907444905001617.

Murshudov, G. N., Skubák, P., Lebedev, A. A., Pannu, N. S., Steiner, R. A., Nicholls, R. A., et al. (2011). REFMAC5 for the refinement of macromolecular crystal structures. *Acta Crystallogr. D Biol. Crystallogr.* 67, 355–367.

doi:10.1107/S0907444911001314.

Musa-Aziz, R., Boron, W. F., and Parker, M. D. (2010). Using fluorometry and ion-sensitive microelectrodes to study the functional expression of heterologously-expressed ion channels and transporters in *Xenopus* oocytes. *Methods* 51, 134–145. doi:10.1016/j.ymeth.2009.12.012.

Musa-Aziz, R., Chen, L.-M., Pelletier, M. F., and Boron, W. F. (2009). Relative CO_2/NH_3 selectivities of AQP1, AQP4, AQP5, AmtB, and RhAG. *Proc. Natl. Acad. Sci. U.S.A.* 106, 5406–5411. doi:10.1073/pnas.0813231106.

Otwinowski, Z., and Minor, W. (1997). [20] Processing of X-ray diffraction data collected in oscillation mode. *Meth. Enzymol.* 276, 307–326. doi:10.1016/S0076-6879(97)76066-X.

Parker, M. D., Qin, X., Williamson, R. C., Toye, A. M., and Boron, W. F. (2012). HCO_3^--independent conductance with a mutant Na^+/HCO_3^- cotransporter (SLC4A4) in a case of proximal renal tubular acidosis with hypokalemic paralysis. *J.Physiol.* 590, 2009–2034.

Phillips, J. C., Braun, R., Wang, W., Gumbart, J., Tajkhorshid, E., Villa, E., et al. (2005). Scalable molecular dynamics with NAMD. *J Comput Chem* 26, 1781–1802. doi:10.1002/jcc.20289.

Preston, G. M., Jung, J. S., Guggino, W. B., and Agre, P. (1993). The mercury-sensitive residue at cysteine 189 in the CHIP28 water channel. *J. Biol. Chem.* 268, 17–20.

Roux, B. (1995). The calculation of the potential of mean force using computer simulations. *Computer Physics Communications* 91, 275–282. doi:10.1016/0010-4655(95)00053-I.

Ryckaert, J.-P., Ciccotti, G., and Berendsen, H. J. C. (1977). Numerical integration of the cartesian equations of motion of a system with constraints: molecular dynamics of n-alkanes. *Journal of Computational Physics* 23, 327–341. doi:10.1016/0021-9991(77)90098-5.

Sui, H., Han, B. G., Lee, J. K., Walian, P., and Jap, B. K. (2001). Structural basis of water-specific transport through the AQP1 water channel. *Nature* 414, 872–878. doi:10.1038/414872a.

Torrie, G., and Valleau, J. (1977). Non-Physical Sampling Distributions in Monte-Carlo Free-Energy Estimation - Umbrella Sampling. *J. Comput. Phys.* 23, 187–199. doi:10.1016/0021-9991(77)90121-8.

Toye, A. M., Parker, M. D., Daly, C. M., Lu, J., Virkki, L. V., Pelletier, M. F., et al. (2006). The human NBCe1-A mutant R881C, associated with proximal renal tubular acidosis, retains function but is mistargeted in polarized renal epithelia. *Am. J. Physiol., Cell Physiol.* 291, C788-801. doi:10.1152/ajpcell.00094.2006.

Virkki, L. V., Franke, C., Somieski, P., and Boron, W. F. (2002). Cloning and functional characterization of a novel aquaporin from *Xenopus laevis* oocytes.

J. Biol. Chem. 277, 40610–40616. doi:10.1074/jbc.M206157200.

Winn, M. D., Ballard, C. C., Cowtan, K. D., Dodson, E. J., Emsley, P., Evans, P. R., et al. (2011). Overview of the CCP4 suite and current developments. *Acta Crystallogr. D Biol. Crystallogr.* 67, 235–242. doi:10.1107/S0907444910045749.

Zhang, L., and Hermans, J. (1996). Hydrophilicity of cavities in proteins. *Proteins* 24, 433–438. doi:10.1002/(SICI)1097-0134(199604)24:4<433::AID-PROT3>3.0.CO;2-F.
